# Characterization of the extrinsic and intrinsic signatures and therapeutic vulnerability of small cell lung cancers

**DOI:** 10.1038/s41392-025-02378-6

**Published:** 2025-09-10

**Authors:** Gui-Zhen Wang, Zheng Wang, Shi-Hao Bai, Yun Tan, Wen-Zhao Zhong, Guo-Gui Sun, Yu-Tao Liu, Bo Pan, Chen Huang, Di Wang, Bei-Bei Sun, Dong-Ni Chen, Bin Zhang, Yong-Chun Zhou, Sheng Li, Xiang-Wei Zhang, Si-Chong Han, Fu-Ying Yang, Xue-Yan Shi, Xiao-Liang Jie, Yu-Ke Shen, Li-Jun Liang, Zhe-Sheng Wen, Li Zhang, Ming-Kun Li, Na Wang, Jin-song Liu, Ying Dong, Man-Li Wang, Yan Wang, Chang-Li Wang, Da-Wei Xie, Ze-Guang Han, Jian-Ming Ying, Chong Chen, Yun-Chao Huang, Hong-Bin Ji, Yuan-Yuan Zhang, Yan Yu, Guang-Biao Zhou

**Affiliations:** 1 State Key Laboratory of Molecular Oncology & Department of Medical Oncology & Department of Pathology, National Cancer Center/National Clinical Research Center for Cancer/Cancer Hospital, Chinese Academy of Medical Sciences and Peking Union Medical College, Beijing, China; 2 Key Laboratory of Systems Biomedicine (Ministry of Education), Shanghai Center for Systems Biomedicine, Shanghai Jiao Tong University, Shanghai, China; 3 Shanghai Institute of Hematology, National Research Center for Translational Medicine, State Key Laboratory of Medical Genomics, Ruijin Hospital Affiliated with Shanghai Jiao Tong University School of Medicine, Shanghai, China; 4 Guangdong Lung Cancer Institute, Guangdong Provincial People’s Hospital (Guangdong Academy of Medical Sciences), Southern Medical University, Guangzhou, China; 5 Department of Chemoradiotherapy, Affiliated Hospital of North China University of Science and Technology, Tangshan, Hebei China; 6 Thoracic Oncology of Harbin Medical University Cancer Hospital, Harbin, China; 7 State Key Laboratory of Oncology in South China, Collaborative Innovation Center for Cancer Medicine, Medical Oncology Department, Sun Yat-Sen University Cancer Center, Guangzhou, China; 8 Department of Lung Cancer, Tianjin Lung Cancer Center, National Clinical Research Center for Cancer, Key Laboratory of Cancer Prevention and Therapy, Tianjin’s Clinical Research Center for Cancer, Tianjin Medical University Cancer Institute and Hospital, Tianjin, China; 9 Department of Thoracic Surgery, the Third Affiliated Hospital of Kunming Medical University, Kunming, China; 10 Key Laboratory of Carcinogenesis and Translational Research (Ministry of Education), Department of Thoracic Oncology, Peking University Cancer Hospital and Institute, Beijing, China; 11 Department of Thoracic Surgery, Shandong Provincial Hospital Affiliated with Shandong First Medical University, Jinan, China; 12 Key Laboratory of Genomic and Precision Medicine, Beijing Institute of Genomics, Chinese Academy of Sciences, China National Center for Bioinformation, Beijing, China; 13 State Key Laboratory of Respiratory Disease, Guangdong Provincial Key Laboratory of Biocomputing, Guangzhou Institutes of Biomedicine and Health, Chinese Academy of Sciences, Guangzhou, China; 14 Department of Medical Oncology, The Second Affiliated Hospital of Zhejiang University School of Medicine, Hangzhou, China; 15 State Key Laboratory of Biotherapy and Cancer Center, West China Hospital, Sichuan University, Chengdu, China; 16 State Key Laboratory of Cell Biology, Innovation Center for Cell Signaling Network, CAS Center for Excellence in Molecular Cell Science, Shanghai Institute of Biochemistry and Cell Biology, Chinese Academy of Sciences; University of Chinese Academy of Sciences, Shanghai, China; 17 Institute of Cancer Research, Henan Academy of Innovations in Medical Sciences, Zhengzhou, China

**Keywords:** Cancer, Lung cancer

## Abstract

Small-cell lung cancer (SCLC), an aggressive neuroendocrine tumor strongly associated with exposure to tobacco carcinogens, is characterized by early dissemination and dismal prognosis with a five-year overall survival of less than 7%. High-frequency gain-of-function mutations in oncogenes are rarely reported, and intratumor heterogeneity (ITH) remains to be determined in SCLC. Here, via multiomics analyses of 314 SCLCs, we found that the *ASCL1*^+^/*MKI67*^+^ and *ASCL1*^+^/*CRIP2*^+^ clusters accounted for 74.38% of the 190,313 SCLC cancer cells from 39 patients, with the *ASCL1*^+^*SOX1*^+^ stem-like cell cluster across SCLC subtypes. The major histocompatibility complex (MHC) class I molecules were expressed at low levels in six and high levels in five cancer cell clusters and were inversely associated with the KI67 expression level. Abnormal splicing of mRNAs was a feature of SCLC, with focal adhesion kinase (FAK) splicing variants identified in 119 (77.3%) of 154 patients. FAK variants exhibited elevated kinase activity, were associated with the worst prognosis, and were sensitive to FAK inhibitors in patient-derived organoids and xenograft models. Eleven high-frequency mutations were identified in addition to *TP53* and *RB1*, and smoking status and tumor stage did not affect microbiota variance in SCLC. Taken together, our data further revealed the complicated ITH and discovered that FAK splicing variants represent high-frequency gain-of-function alterations in oncogene in SCLC and potential therapeutic targets for this recalcitrant cancer.

## Introduction

Small cell lung cancer (SCLC), a highly aggressive malignancy that accounts for 15% of all lung cancer cases and kills 270,000 patients annually worldwide,^1^ is rarely treated with surgery, resulting in a lack of specimens for in-depth dissection of disease pathogenesis. In SCLC, loss-of-function mutations have been detected in tumor suppressors, including *TP53* and *RB1*, but high-frequency gain-of-function mutations in oncogenes have rarely been identified.^2–6^ On the basis of the expression of the transcription factors Achaete-scute homolog 1 (ASCL1, A), Neurogenic differentiation factor 1 (NEUROD1, N), POU class 2 homeobox 3 (POU2F3, P), and Yes-associated protein 1 (YAP1, Y)^7^ and an inflamed gene signature, SCLC is molecularly classified as SCLC-A, N, P, Y, and I.^6–8^ Increased intratumoral heterogeneity (ITH) and an immunosuppressive tumor microenvironment (TME) have been detected across these subtypes.^9–11^ Although immunotherapy has been used, the 5-year survival rate for patients with SCLC at all stages remains <7%.^12^ Therefore, multiomics studies are still needed to identify high-frequency gain-of-function alterations in oncogenes and molecular subtypes for treatment optimization.

SCLC is closely associated with environmental risk factors such as tobacco smoke, air pollution (haze), tin, asbestos, radon, etc.^13^ The two most important risk factors for SCLC^13,14^ are tobacco smoke and haze (smohaze), which contain more than 30 lung carcinogens, including polycyclic aromatic hydrocarbons (PAHs), such as benzo(a)pyrene (BaP), and *N*-nitrosamines, such as 4-(methylnitrosamino)-1-(3-pyridyl)-1-butanone (NNK).^15,16^ Carcinogens have been shown to increase the risk of alternative splicing,^17^ a process by which splice sites are differentially utilized to produce different mRNA isoforms, contributing to the abnormal activation of oncogenic pathways. In SCLC, alternative splicing events (ASEs) have been detected in several genes,^2,18^ and splicing factors^5,19^ are abnormally expressed. However, the ASE profiles of SCLCs have not been characterized, and the roles of ASEs in SCLC pathogenesis and treatment regimens remain to be systematically explored.

Carcinogens usually cause characteristic mutations in the genome, providing clues for tracing the initiators of tumorigenesis. For example, BaP induces C:G→A:T transversions^20,21^ and the so-called Signature 4 when the 5’ and 3’ sequence context of the mutated base is considered,^22^ whereas NNK mainly induces C:G→T:A transitions.^23^ In smokers with SCLC, the number of C:G→A:T transversions is 3.73 mutations/Mb, which is lower than that in smokers with lung adenocarcinoma (LUAD; 4.88 mutations/Mb) or lung squamous cell carcinoma (LUSC; 4.19 mutations/Mb).^24^ This degree of transversion is also low in head and neck squamous cell carcinoma,^25^ suggesting that the proximal airway might be exposed to tobacco smoke for a shorter time than the distal airway is and thus has lower mutation rates. However, the specific carcinogenic compounds responsible for SCLC remain to be determined, and microbiome profiles in this disease need to be determined.

In this study, we conducted single-cell RNA sequencing (scRNA-seq), bulk RNA-seq, ASE assessment, chemokine analysis, 16S rRNA gene sequencing, and whole-exome sequencing (WES) on samples from 314 patients with SCLC (Supplementary Fig. 1). Our results shed light on the ITH and ASE profiles of SCLCs. Splicing alternatives of *protein tyrosine kinase 2* (*PTK2*)/*focal adhesion kinase* (*FAK*) were detected in 119 (77.3%) of the 154 SCLCs. These variants are associated with poor prognosis, exhibit elevated tyrosine kinase activity and are sensitive to FAK inhibitors, representing high-frequency gain-of-function alterations in oncogenes and potential therapeutic targets for SCLC.

## Results

### Description of the SCLC cohort

To enable in-depth investigation of SCLC tumorigenesis and therapeutic vulnerabilities, we collected paired tumor and normal specimens from 314 patients, sourced from 10 hospitals spanning 8 cities in China. Among these patients, 300 (95.5%) were diagnosed with SCLC, and 14 (4.5%) had combined SCLC. Demographically, 237 (75.5%) patients were male, and 77 (24.5%) were female. A total of 150 (47.8%) were under 60 years of age, and 164 (52.2%) were 60 years or older. Among these patients, 208 (66.2%) were smokers, and 88 (28.0%) were nonsmokers. In terms of disease stage, 125 (39.8%) patients were classified as stage IA-IIB, and 154 (49.0%) were classified as stage IIIA-IV (Supplementary Table 1).

**Fig. 1:**
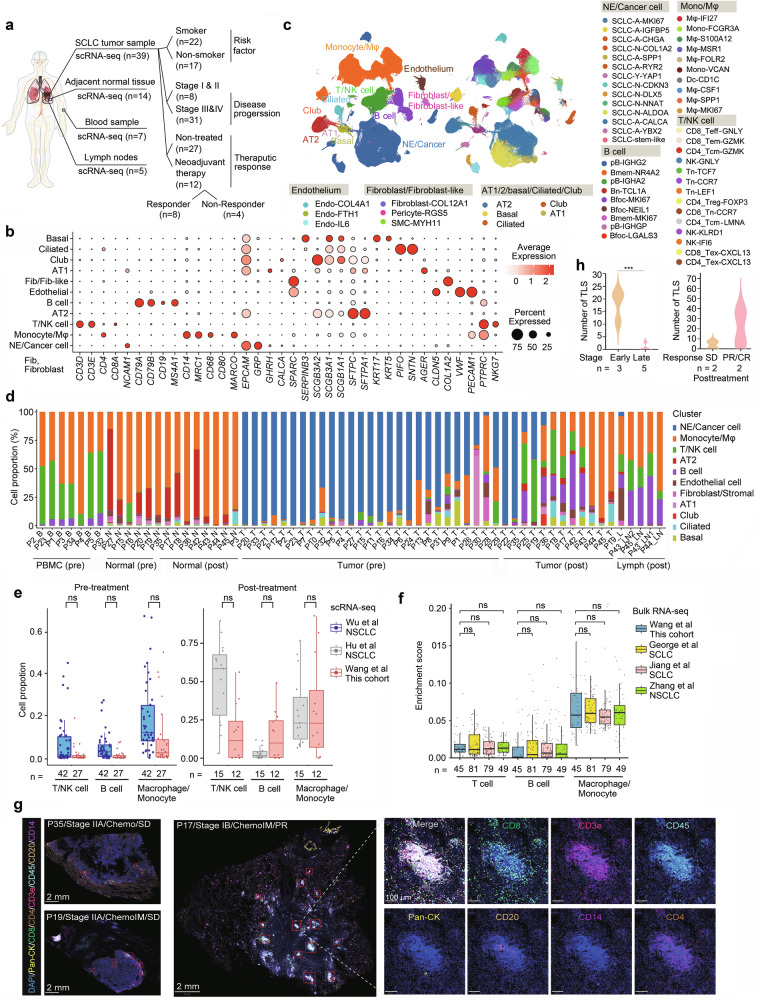
Microenvironment landscape of small cell lung cancer (SCLC). **a** Schematic representation of the transcriptomic study design, utilizing a total of 65 samples from 39 patients with SCLC for single-cell RNA sequencing. **b** Dot plot of selected marker genes in each cell lineage. Dot size and color indicate the fraction of expressing cells and normalized expression levels, respectively. **c** UMAP visualization of expression profile clusters for cancer and immune cells within the tumor microenvironment, identifying 7 major cell types (left panel) and 58 major subtypes (right panel). **d** Bar plots illustrating the distribution of the 7 major cell types in each sample, categorizing samples as tumor samples (pre- or posttherapy), adjacent normal tissues (pre- or posttherapy), peripheral blood mononuclear cells (PBMCs), and lymph nodes. **e** Comparison of the ratios of T/NK cell, Mφ/monocyte, and B cell in the tumor tissues of patients with NSCLC (Wu et al.^28^ and Zhang et al.^29^) and SCLCs (this cohort, Wang et al.) via scRNA-seq analysis. Data were obtained from the scRNA-seq datasets GSE148071^28^ and GSE207422.^29^ **f** Comparison of the relative enrichment of T cell, Macrophage/monocyte, and B cell in the bulk RNA-seq data of this cohort (Wang et al.), George et al.,^2^ Zhang et al.^125^ and Jiang et al.^5^ cohorts. The enrichment scores for T cells, macrophages/monocytes, and B cells were calculated via the Xcell algorithm. **g** Tertiary lymphoid structures (TLSs) in patients receiving neoadjuvant therapy. Images of three patients are shown, and patient characteristics are listed within the images. Pan-CK, pancytokeratin. **h** Quantification of TLSs in **g**. *P* value, Student’s *t* test. ***, *P* < 0.001

To comprehensively investigate intrinsic and extrinsic alterations in SCLC, we performed scRNA-seq on 65 samples (39 tumors, 14 adjacent normal lung tissues, 7 peripheral blood mononuclear cells, and 5 lymph nodes) from 39 patients (Fig. 1a), bulk RNA-seq of 45 tumor-normal tissue pairs, 16S rRNA-seq to assess microbial communities in 53 tumor-normal pairs, WES of 111 tumor-normal pairs, single-cell spatial proteomics of 12 formalin-fixed paraffin-embedded (FFPE) samples, cytokine/chemokine profiling of serum from 62 SCLC patients and 56 healthy donors, and ASE validation of an independent set of 136 SCLC tumors (Supplementary Table 1, Supplementary Fig. 1).

### Major cell populations in SCLC

To define major cell populations in SCLC, single-cell transcriptomic profiling analyses were performed in this study. For scRNA-seq, 432,959 single cells were obtained from 27 untreated patients and 12 treated patients after quality control (Materials and Methods), and 7 major cell types and 58 subtypes were identified via the selection of marker genes in each cell lineage (Fig. 1b, c, Supplementary Fig. 2a, b). The major cell types included endothelial cell, fibroblast/fibroblast-like cell, alveolar type I/II (AT1/2)/basal/ciliated/club cell, neuroendocrine (NE)/cancer cell, macrophage (Mφ)/monocyte, B cell, and T/NK cell (Fig. 1c, Supplementary Fig. 2a). As previously described,^9^ cluster NE cell was considered to represent SCLC cancer cell. We found that among the 27 pretreatment tumors, 19 (70.4%), 7 (25.9%), and 1 (3.7%) lung tumor had fewer (<10%), moderate (10–50%), and more (>50%) immune cells, respectively (Fig. 1d and Supplementary Table 2), suggesting a heterogeneous immune microenvironment across patients. Cancer cells were also detected in blood samples (Supplementary Table 2), which is consistent with previous reports.^26,27^ Compared with normal lung tissues, tumor samples had fewer AT2 cells and Mφs, along with an expansion of cancer cells (Supplementary Fig. 2c). B cells were significantly lower in stage III and IV SCLC samples than in stage I and II SCLC samples in both the pre- and posttreatment tumor samples (Supplementary Fig. 2d). Among the 12 patients who were treated with chemotherapy (n = 4) or chemoimmunotherapy (n = 8: etoposide/cisplatin plus an anti-PD-L1 antibody), 4, 4, 3, and 1 patient(s) achieved complete response (CR), partial response (PR), stable disease (SD), and progressive disease (PD), respectively. We compared the cell composition between responders and nonresponders and observed significantly greater proportions of Mφs and basal and ciliated cells but significantly fewer cancer cells in responders (Supplementary Fig. 2e).

### Comparative analysis of immune cells in SCLC and non-small cell lung cancer

To test the potential differences in immune infiltration between SCLC and non-small cell lung cancer (NSCLC), we compared the ratios of immune cells in SCLC tumor samples from this cohort with those in NSCLC samples from Chinese ancestry.^28,29^ Compared with NSCLC samples, pretreated SCLC samples had slightly lower, although not statistically significant, fractions of T/NK cell, B cell, and Mφ, while posttreatment SCLC samples had slightly higher B cell fractions than did NSCLC samples (Fig. 1e). Deconvolution analyses of bulk RNA-seq data from different datasets revealed no significant differences in T cell, B cell, or Mφ levels between SCLC and NSCLC patients (Fig. 1f).

### Spatial distribution of immune cells in SCLC

To further elucidate the spatial distribution of SCLC and immune cells in SCLC tumor samples, we performed a spatial proteomics assay at single-cell resolution via PhenoCycler-Fusion 2.0 (Supplementary Fig. 3a). We found that tumor tissues had fewer infiltrating immune cells than normal lung tissues did, and tumors from patients with early-stage SCLC presented more granzyme B^+^/CD8^+^ T cells than those from patients with late-stage SCLC did (Supplementary Fig. 3b-d). Posttreatment, CR and PR patients presented higher numbers of these immune cells than patients with SD did (Supplementary Fig. 3e, f). Moreover, we detected tertiary lymphoid structures (TLSs) in tumors, revealing more TLSs in early-stage patients than in late-stage patients and nonresponders to therapy (Fig. 1g, h). These TLSs were positive for CD45, CD8, CD3, CD4 and CD20 but weak for CD14 (Fig. 1g).

### Immune cells in smoking and nonsmoking patients

We analyzed differences in various cell populations between smokers (n = 22) and nonsmokers (n = 17). Although there were no significant differences in the individual cell populations between the two groups, a distinct differential expression pattern of genes in various cell subtypes was noted. For example, pathways related to pyridine-containing compound metabolism and mitochondrial electron transport from cytochrome C to oxygen were enriched, whereas pathways related to DNA damage by the p53 class mediator and the regulation of epidermal cell differentiation were suppressed in cancer cells from smokers compared with those from nonsmokers (Supplementary Fig. 4a). Additionally, Mφ from smokers presented higher levels of *apolipoprotein E* (*APOE*) and *Secreted Phosphoprotein 1* (*SPP1*) and lower levels of human leukocyte antigen (HLA) class II DR beta 6 (*HLA*-*DRB6*) than those from nonsmokers did (Supplementary Fig. 4b).

### Alterations in immune cells in the SCLC microenvironment

The SCLC TME has previously been characterized in non-immune (CD45^-^) cells,^9^ circulating tumor cells (CTCs),^26^ CTC-derived xenografts,^10^ and limited patient tumor/adjacent normal tissue samples (n=11).^11^ We further characterize the SCLC TME in our cohort.

### Macrophage

**Fig. 2:**
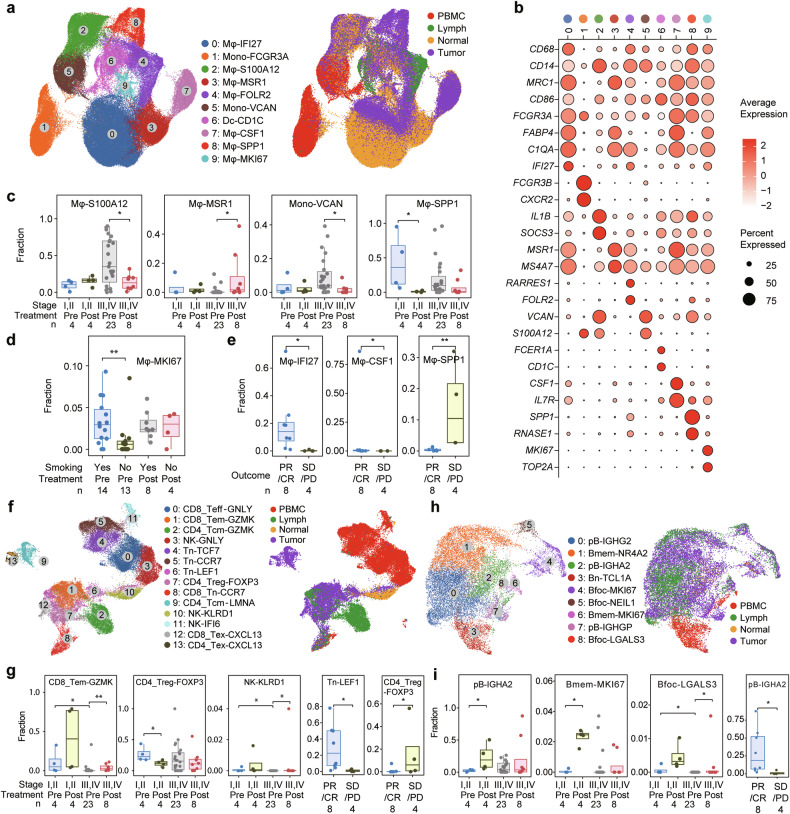
Immune cells in the microenvironment of SCLC. **a** Analysis of macrophages (Mφs)/monocytes. A total of 7 Mφ subtypes, 2 monocyte subtypes and 1 dendritic cell subtype were identified (left panel). The distribution of cells across different sample types is shown in the right panel. **b** Dot plot of selected gene expression in each cell lineage. Dot size and color indicate the fraction of expressing cells and normalized expression levels, respectively. **c-e** Boxplots representing the relative proportions of each Mφ/monocyte population. Comparisons were made among different stages classified by TNM stage (**c**), smoking status (**d**), and outcomes of neoadjuvant therapy (**e**). **f** Analysis of T/NK cells. A total of 14 subtypes of T/NK cells were identified (left panel). The distribution of cells in different sample types is shown in the right panel. **g** Boxplots representing the relative proportions of each T/NK cell subtype. Comparisons were made among different stages and outcomes of neoadjuvant therapy. **h** Analysis of B cells. A total of 9 subtypes of B cells were identified (left panel). The distribution of cells across different sample types is shown in the right panel. **i** Boxplots representing the relative proportions of each B cell subtype. Comparisons were made among different stages and outcomes of neoadjuvant therapy. *P* values in (**c**, **d**, **e**, **g**, and **i**) were determined via the Wilcoxon rank-sum test. *P < 0.05, **P < 0.01

We investigated the immune cell subtypes residing within the intricate microenvironments of patients with SCLC. We identified 7 distinct Mφ subtypes, 2 monocyte subtypes, and 1 DC cluster (Fig. 2a, b, and Supplementary Table 3). Mφ subtypes vary across sample types, tumor stages, and neoadjuvant therapies. Specifically, Mφ-*IFI27* (for *Interferon Alpha* *Inducible Protein27*) and Mφ-*MSR1* (*Macrophage* *Scavenger* *Receptor1*) were identified primarily in normal adjacent samples, whereas most monocytes were detected in peripheral blood mononuclear cell (PBMC) samples (Fig. 2a and Supplementary Fig. 4c). Postneoadjuvant therapy, Mφ-*S100A12* (*S100 Calcium Binding Protein A12*), Mono-*VCAN* (*Versican*), and Mφ-*SPP1* decreased, whereas Mφ-*MSR1* increased (Fig. 2c). Mφ-*MKI67* (*marker of proliferation Ki-67*) levels were significantly greater in smokers than in nonsmokers within the untreated population (Fig. 2d). Additionally, Mφ-*IFI27* and Mφ-*CSF1 (colony stimulating factor 1)* levels were elevated in patients who achieved PR/CR, whereas Mφ-*SPP1* was lower in these patients than in those with SD/PD (Fig. 2e). Mφ-*SPP1*, which has been linked to angiogenesis and tumor progression,^30^ was further characterized by delineating its transcriptomic features. We revealed that in Mφ-*SPP1*, genes involved in receptor-mediated endocytosis and chemokine responses, such as *APOE*, *mannose receptor C-type 1* (*MRC1*), and *C-X-C motif chemokine ligand 8* (*CXCL8*), were significantly upregulated (Supplementary Fig. 4d and Supplementary Table 4).

#### T/NK cell

Our analysis identified 14 distinct T/NK cell subsets, including naïve T (Tn) cell Tn-*TCF7* (*Transcription Factor 7*), Tn-*CCR7* (*C-C Motif Chemokine Receptor 7*) and Tn-*LEF1* (*Lymphoid Enhancer Binding Factor 1*), central memory Tcm-*GZMK* (*Granzyme K*) and Tcm-*LMNA* (*Lamin A/C*), effector Teff-*GNLY* (*Granulysin*), effector memory Tem-*GZMK*, exhausted CD4^+^ and CD8^+^ Tex-*CXCL13* (*C-X-C Motif Chemokine Ligand 13*), regulatory Treg-*FOXP3* (*Forkhead Box P3*), NK cell NK-*GNLY*, NK-*KLRD1* (*Killer Cell Lectin Like Receptor D1*) and NK-*IFI6* (*Interferon Alpha Inducible Protein 6*) clusters (Fig. 2f, Supplementary Fig. 2b, and Supplementary Table 3). In tumor samples from this cohort, we identified 84 NK cells, accounting for 0.52% of the total T/NK cells and 0.03% of all tumor cells. Additionally, IHC analysis of FFPE samples from three patients with untreated SCLC revealed CD94 (an NK marker) positivity in 3,548 (0.36%) out of 973,346 tumor cells, as quantified by Aipathwell software (Supplementary Fig. 4e). The CD8_Tem-*GZMK* and NK-*KLRD1* levels increased after neoadjuvant therapy, whereas the CD4_Treg-*FOXP3* levels decreased (Fig. 2g).

#### B cell

We identified 9 distinct B cell subclusters, including naïve B (Bn) cell Bn-*TCL1A* (*TCL1 Family AKT Coactivator A*), memory B (Bmem) cell types Bmem-*NR4A2* (*Nuclear Receptor Subfamily 4 Group A Member 2*) and Bmem-*MKI67*, follicular B (Bfoc) Bfoc-*NEIL1* (*Nei Like DNA Glycosylase 1*), Bfoc-*LGALS3* (*Galectin 3*), and Bfoc-*MKI67* subtypes, and plasma cell pB-*IGHA2* (*Immunoglobulin Heavy Constant Alpha 2*), pB-*IGHG2* (*Immunoglobulin Heavy Constant Gamma 2*), and pB-*IGHGP* (*Immunoglobulin Heavy Constant Gamma P*) subtypes (Fig. 2h and Supplementary Table 3). Following neoadjuvant therapy, the levels of pB-*IGHA2*, Bmem-*MKI67*, and Bfoc-*LGALS3* increased, with higher pB-*IGHA2* levels in patients who achieved PR or CR (Fig. 2i). These results suggest that B cells may play an antitumor role in SCLC, which is consistent with their roles in lung adenocarcinoma.^31^

### Heterogeneity of SCLC cells

#### Cell clusters

**Fig. 3:**
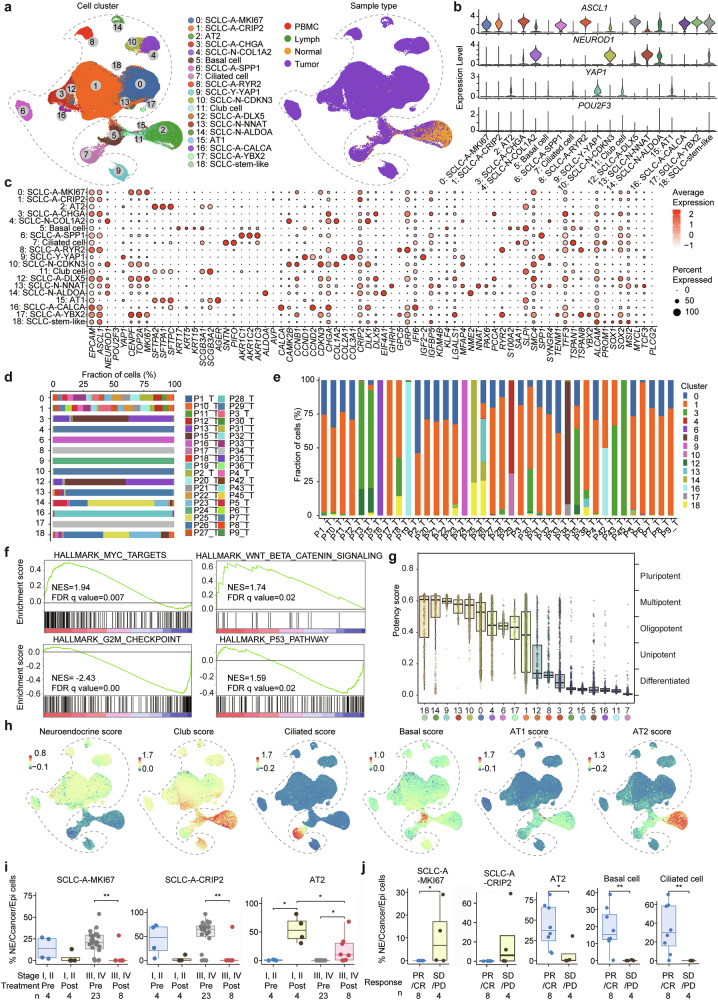
Cancer cells and cancer stem-like cells in SCLC. **a** Analysis of cancer cells and nonimmune normal cells identified 19 cell clusters (left panel). The distribution of cells in different sample types is shown in the right panel. **b, c** Expression of feature genes in the cell clusters. The expression of *ASCL1*, *NEUROD1*, *YAP1*, and *POU2F3* is shown in a violin plot (**b**). The marker genes of each cluster are shown in dot plots (**c**). Dot size and color indicate the fraction of expressing cells and normalized expression levels, respectively. **d** Relative fractions of tumor samples in the 14 cancer cell clusters. **e** Distribution of distinct tumor cell clusters across the 39 tumor samples. **f** Gene set enrichment analysis (GSEA) plots for the indicated gene sets in the stem-like cell cluster and the other cancer cell cluster. **g** Potency and differentiation states of the 19 cell clusters evaluated by the CytoTrace algorithm. **h** Enrichment scores of potential cell origins for SCLC in lung tissues. The top 100 genes in neuroendocrine, club, ciliated, basal, AT1, and AT2 cells were used for enrichment analysis. **i, j** Comparisons of the indicated cell clusters at different stages (**i**) and outcomes of neoadjuvant therapy (**j**). *P* values in panels h and i were determined via the Wilcoxon rank-sum test. *P < 0.05; **P < 0.01

To elucidate the intricate features of cancer cells, we analyzed the nonhematopoietic cells of the tumor samples and identified 19 cellular clusters, including 5 noncancerous clusters: AT1, AT2, basal, ciliated, and club cells (Fig. 3a, b). A total of 190,313 cancer cells were obtained, which were divided into 14 subclusters: C0: SCLC-A-*MKI67*, C1: SCLC-A-*CRIP2* (*Cysteine Rich Protein 2*), C3: SCLC-A-*CHGA* (*Chromogranin A*), C4: SCLC-N-*COL1A2* (*Collagen Type I Alpha 2 Chain*), C6: SCLC-A-*SPP1*, C8: SCLC-A-*RYR2* (*Ryanodine Receptor 2*), C9: SCLC-Y-*YAP1*, C10: SCLC-N-*CDKN3* (*Cyclin Dependent Kinase Inhibitor 3*), C12: SCLC-A-*DLX5* (*Distal-Less Homeobox 5*), C13: SCLC-N-*NNAT* (*Neuronatin*), C14: SCLC-N-*ALDOA* (*Aldolase, Fructose-Bisphosphate A*), C16: SCLC-A-*CALCA* (*Calcitonin Related Polypeptide Alpha*), C17: SCLC-A-*YBX2* (*Y-Box Binding Protein 2*), and C18: SCLC-stem-like cell (SLC) (Fig. 3b, c).

#### Dominant cancer cell populations

The number of C1 and C0 clusters reached 100,754 (52.94%) and 40,807 (21.44%) of all the cancer cells (Supplementary Table 5), representing the two most dominant cancer cell populations. The median proportions of C1 and C0 in patients were 65.52% (0–100%) and 18.59% (0–53.78%), respectively (Supplementary Table 5, Fig. 3d). Patient #44, who achieved CR after chemoimmunotherapy, had no detectable cancer cells posttreatment; therefore, no data for this patient are shown in Fig. 3d. Additionally, most tumor samples contained multiple cancer cell clusters, and both SCLC-A and SCLC-N-cell populations were detected in 28 (71.79%) of the 39 samples (Fig. 3e). *Hepatocyte nuclear factor 4 alpha* (*HNF4A*) is a newly emerging signature for neuroendocrine carcinomas, particularly gastrointestinal neuroendocrine carcinoma.^32^ However, the expression of *HNF4A* was relatively low in cancer cells according to our scRNA-seq data (Supplementary Fig. 5a) and bulk RNA-seq data (Supplementary Fig. 5b). In addition, compared with normal epithelial cells, cancer cell clusters displaced a greater copy number variation (CNV) burden, which is consistent with a malignant phenotype (Supplementary Fig. 5c).

#### The stem-like cell population

The C18 cluster expressed genes associated with stem cell and cancer-initiating cell features, including *activated leukocyte cell adhesion molecule* (*ALCAM*)/*CD166*,^33^ *Musashi 2* (*MSI2*),^34^ *SRY-box transcription factor 2* (*SOX2*),^35^ *SOX1*,^36^ *insulin-like growth factor binding protein 5* (*IGFBP5*),^37^ *L-Myc* (*MYCL*),^38^ *prominin-1* (*PROM1*)/CD133,^39^ *transcription factor 3* (*TCF3*),^40^ and the neuroendocrine marker *chromogranin A* (*CHGA*) (Fig. 3c). Gene set enrichment analysis (GSEA) revealed that, compared with other types of cancer cells, SLCs presented significant enrichment of MYC targets and WNT-β-catenin pathway genes but inhibited the cell cycle G2M checkpoint and p53 pathway (Fig. 3f). These results provide evidence for further functional studies to validate the biological significance of the identified pathways. Compared with other cell clusters, SLCs presented the highest pluripotency score (Fig. 3g), as assessed via the computational framework CytoTRACE.^41^ The SLCs included 1771 cells, accounting for 0 to 25.78% (median, 0.05%) of all cancer cells in each patient (Supplementary Table 5). In a stage IV patient (#P26) who died three days after diagnosis, SLC accounted for 569 (25.78%) of the 2207 cancer cells. In two patients who received neoadjuvant therapy, SLCs represented >10% of the posttreatment tumor samples. SLCs were identified in 27 (69.2%) of the 39 patients, including 23, 3, and 1 patient with SCLC-A, SCLC-N, and SCLC-Y subtypes, respectively (Supplementary Table 5). SLCs were positive in 16 (57.1%) of 28 males and 11 (100%) of 11 females and in 12 (54.5%) of 22 smokers and 15 (88.2%) of 17 nonsmokers (Supplementary Table 5). The stemness of this cluster warrants further investigation.

We performed enrichment analyses of identity gene markers for epithelial cells via the “weighted-nearest neighbor” method^42^ and found that almost all cancer cells, except for SCLC-*YAP1* cells, exhibited neuroendocrine enrichment scores (Fig. 3h). Moreover, clusters 1, 13, 17, 8, and 4 of SCLC-A and SCLC-Y cells presented moderate-to-strong club cell and weak basal cell scores (Fig. 3h), suggesting a potentially diverse origin of SCLC. However, studies have shown that during lung development, Notch signaling induces a neuroendocrine-to-nonneuroendocrine switch by blocking precursor differentiation into neuroendocrine cell, and promoting neuroendocrine-to-club cell transformation through RE1-silencing transcription factor (REST) and YAP1.^43,44^ Hence, these results suggest that SCLC may still develop from a common progenitor cell.

#### Dynamics of SCLC-A-MKI67 and SCLC-A-CRIP2 clusters

We analyzed distinct SCLC cell clusters across various disease stages and found that the SCLC-A-*MKI67* and SCLC-A-*CRIP2* fractions were relatively high in both stages I–II and III–IV and were significantly reduced upon neoadjuvant therapy, accompanied by a notable increase in AT2 cells. In pretreatment samples, the SCLC-A-*MKI67* cluster accounted for 20.93% (range, 0.02–53.78%) of all cancer cells in each patient; in posttreatment samples, this cluster was eradicated (Fig. 3i), confirming the clinical response to initial treatment. The SCLC-A-*CRIP2* cluster accounted for 69.08% (range, 0–96.82%) of the pretreatment samples, and 33.33% (0–100%) of all cancer cells in the posttreatment samples (Fig. 3i), suggesting its potential role in drug resistance and relapse. Upon neoadjuvant therapy, the fraction of SCLC-A-*MKI67* cells was lower in patients who achieved PR/CR than in those who achieved SD/PD, and the fraction of non-cancer cells was greater in the PR/CR group than in the SD/PD group (Fig. 3j).

### Heterogenous expression of major histocompatibility complex components

**Fig. 4:**
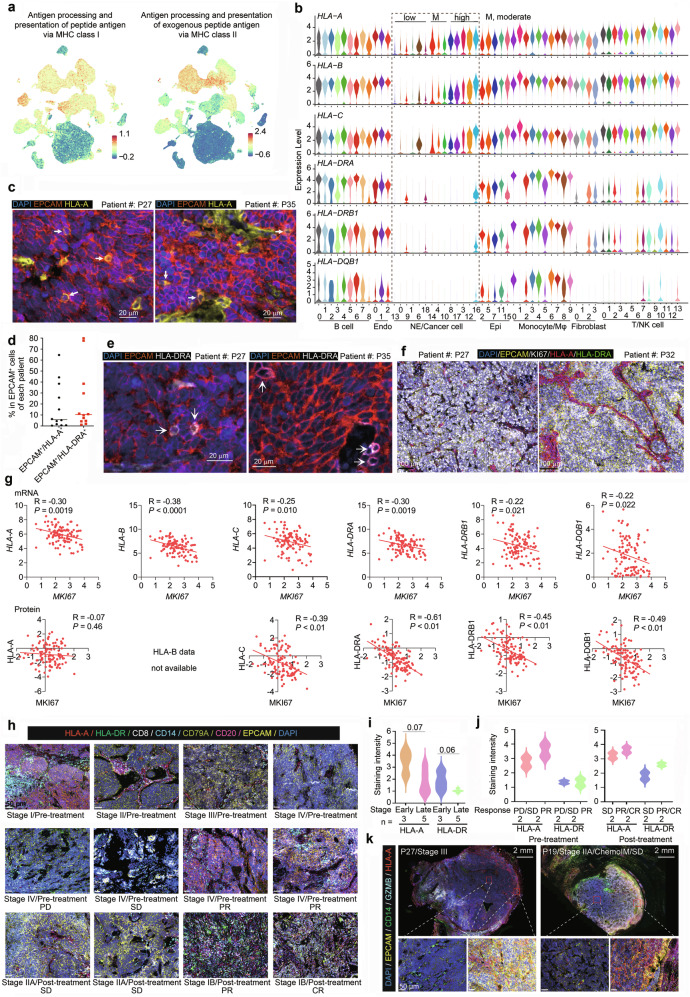
Antigen processing and presentation (APP) machinery in SCLC. **a** UMAP displaying the enrichment of genes related to APP via MHC-I and MHC-II. The enrichment scores are visualized via color coding. **b** The expression of MHC molecules in the indicated cell clusters. **c**–**e** The expression of HLA-A and HLA-DRA on EPCAM^+^ cancer cells. Two representative images from two patient samples are shown (**c**, **e**), and 12 patient samples were tested via PhenoCycler-Fusion 2.0. The ratios of EPCAM^+^HLA-A^+^ and EPCAM^+^HLA-DRA^+^ EPCAM^+^ cells in each patient are shown in (**d**). **f** The expression of HLA-A and HLA-DRA in EPCAM^+^KI67^+^ cancer cells. Two representative images from two patient samples are shown, and 12 patient samples were tested via PhenoCycler-Fusion 2.0. **g** The correlation between the expression levels of KI67 and MHC components in SCLC (n = 107). The data were obtained from Liu et al.^6^ *P* values, Pearson correlation test. **h**–**j** HLA-A/HLA-DR expression in cancer and immune cells (**h**), and the staining intensity of HLA-A and HLA-DR on cancer and immune cells was obtained via QuPath (**i**, **j**). Patient characteristics are listed at the bottom of each image. *P* value, Student’s *t* test. **k** HLA-A expression in cancer and immune cells localized at the center and border of the tumors. Enlarged signals are shown in the lower panels

We analyzed the antigen processing and presentation (APP) pathway in SCLC and found that APP through major histocompatibility complex class I (MHC-I) and MHC-II was markedly suppressed in most cancer cells (Fig. 4a), which is consistent with previous reports.^45^ We analyzed MHC-I scores in each cell cluster (Materials and Methods) and reported that the expression of *HLA-A*, *HLA-B*, and *HLA-C* was low in cancer cell clusters 13, 0, 9, 1, 6, and 18; moderate in clusters 14, 4, and 10; and high in clusters 8, 17, 3, 12, and 16 (Fig. 4b), indicating the heterogeneous expression of MHC-I in SCLC cell populations. The total number of MHC-I-high clusters was 19,191, accounting for 10.08% of all SCLC cancer cells (Supplementary Table 5). To validate the above findings, we analyzed our spatial proteomics data and found that a proportion of EPCAM^+^ cancer cells (median, 10,212; range: 150–167,784) also expressed HLA-A (Fig. 4c), with a median ratio of 5.79% (range, 0.3–64.63%; Fig. 4d and Supplementary Table 5). A proportion of EPCAM^+^ cancer cells (median, 21,286; range, 129–197,205) expressed HLA-DRA (Fig. 4e), with a median ratio of 10.28% (range, 1.08–79.6%; Fig. 4d and Supplementary Table 5). However, EPCAM^+^KI67^+^ cells were negative for HLA-A and HLA-DRA (Fig. 4f). We analyzed the potential associations between MHC components and marker genes of MHC-I-low or high clusters via SCLC proteomic data^6^ and found that the expression levels of MKI67 were inversely associated with the expression levels of MHC-I and MHC-II molecules (Fig. 4g). Hence, the heterogeneity of MHC expression in SCLC cell clusters and the role of MKI67 in the regulation of MHC-I and MHC-II warrant further investigation.

We analyzed MHC molecules in 50 SCLC and 1351 other cancer cell lines using Cancer Cell Line Encyclopedia (CCLE) data (https://sites.broadinstitute.org/ccle/) and reported that the expression of MHC-I and MHC-II components was lower in SCLC lines than in other cancer cell lines (Supplementary Fig. 5d). A comparative study of SCLC tumors harvested before and after neoadjuvant therapy revealed that APP enrichment scores via MHC-I/II increased after neoadjuvant therapy (Supplementary Fig. 5e), with higher scores in patients who achieved a PR/CR than in those with SD/PD (Supplementary Fig. 5f).

The expression of MHC-II molecules was low in almost all cancer cell clusters (Fig. 4b). MHC-II molecules, particularly HLA-DQB1, were low in almost all T/NK cell clusters, whereas HLA-DRA and HLA-DRB1 were low in T/NK cell clusters 1 and 8. HLA-DRA, DRB1, and DQB1 were also low in cluster 1 of Mφs/monocytes (Fig. 4b). Cell‒cell interaction analyses via the CellChat method (https://github.com/sqjin/CellChat) revealed that among the 39 patients, the interaction probabilities between cancer cell and T/NK cell/Mφ via MHC-I and MHC-II were low (Supplementary Fig. 5g). Single-cell spatial proteomic analysis revealed that both HLA-A and HLA-DR levels were higher in tumor tissues from patients with early-stage SCLC than in those from patients with late-stage SCLC (Fig. 4h, i). Patients with higher HLA-A/HLA-DR expression in pretreatment cancer cells responded better to neoadjuvant therapy, and HLA-A/HLA-DR levels were higher in responders than in nonresponders (Fig. 4h, j). Cancer cells located at the tumor center stained weaker for HLA-A, whereas those at the tumor border had stronger staining and were surrounded by immune cells (Fig. 4k).

#### Transcriptomic profiles of SCLC

Since scRNA-seq analyzes approximately 2000 genes per cell by sequencing 150 bp from the 3’ end, structural changes in corresponding genes were not captured. Therefore, we performed bulk RNA-seq on tumor-normal samples from 45 patients with SCLC to analyze the transcriptomics and potential ASEs. Among these patients, 40 (88.9%) were male, and 36 (80.0%) were smokers (Supplementary Table 1). Compared with those in normal lung tissues, 2893 genes were upregulated, and 1213 genes were downregulated in tumor tissues (Supplementary Table 6). The genes whose expression was most frequently upregulated in tumor tissues were enriched in proliferation pathways, including nuclear division, DNA replication, and the cell cycle, whereas the genes whose expression was downregulated were involved in wound healing and cytokine‒cytokine receptor interaction pathways (Supplementary Fig. 6a). GSEA revealed that, in tumor tissues, the upregulated genes were enriched in the neuropeptide signaling pathway and neuronal fate commitment, whereas the downregulated genes were related to T-cell-mediated immunity and cytotoxicity (Supplementary Fig. 6b). Additionally, 793 genes were upregulated and 593 were downregulated in the tumor tissues of smokers compared with nonsmokers (Supplementary Table 6). The upregulated genes were enriched in cellular processes involved in reproduction in multicellular organisms and nicotine addition, whereas the downregulated genes were enriched in epidermal development, protein digestion and absorption, and other processes (Supplementary Fig. 6c).

#### Tumor microenvironment characteristics

The composition of the TME was analyzed via the xCell method,^46^ and the results revealed that, compared with normal lung tissues, tumor tissues presented lower immune scores and lower T/B cell and T helper cell activation scores (Supplementary Fig. 7a). Nevertheless, 33 (73.3%) of the 45 tumors were clustered as “cold” tumors, and 12 (26.7%) were categorized as “hot” tumors, based on their TME characteristics.^47^ According to a 50-gene expression-based NE score,^48^ 29 (64.4%) and 16 (35.6%) patients had NE and low-NE SCLC, respectively (Supplementary Fig. 7a). We found that 27 (93.1%) of the 29 NE SCLC tumors presented cold immune characteristics, and 10 (62.5%) of the 16 low-NE SCLC tumors presented hot immune characteristics (*P* = 1.2e-4, Fisher’s exact test) (Supplementary Fig. 7a), which is consistent with previous reports.^11^

Bulk RNA-seq analysis confirmed the downregulation of MHC-I/II genes in tumor samples (Supplementary Fig. 7b), as revealed by scRNA-seq (Fig. 4). Compared with normal controls, tumor samples had fewer fibroblasts, endothelial cells, neutrophils, AT2 cells, and monocytes but more NE, Treg, Th2 and Th1 cells (Supplementary Fig. 7c), which have been previously shown to be enriched^49^ but functionally inhibited in NSCLCs.^50^ Compared with normal lung tissues, tumor tissues presented elevated *SPP1*, *IL36RN*, *CXCL13*, *CXCL14*, *MIF*, *LTB4R*, and other cytokines/chemokines (Supplementary Fig. 7d). To validate this, the serum concentrations of CXCL13, CXCL14, and CCL20 were measured in 62 patients with SCLC and 56 age-, sex-, and smoking-matched healthy donors. The concentrations of CXCL13 and CXCL14 were significantly greater in patients with SCLC than in healthy donors (Supplementary Fig. 7e). Although *CCL20* mRNA expression was lower in tumor tissues than in normal control tissues (Supplementary Fig. 7d), the CCL20 serum concentration was higher in patients than in matched healthy donors (Supplementary Fig. 7e), confirming that serum chemokines originate from various cells across different tissues and organs.^51^

#### Structural abnormalities and high-frequency splicing variants

We used the STAR-Fusion^52^ and FusionInspector tools to detect fusion genes and excluded those found in normal samples and obtained 32 candidates, of which nine (*ARID1B-ZDHHC14*, *CSNK1D-CCDC57*, *CTNNBIP1-CLSTN1*, *DNER-PID1*, *EIF4G3-ECE1*, *MED20-USP49*, *PSMB7-NR6A1*, *TIRAP-DCPS*, and *TPD52L2-DNAJC5*) were reported in a previous study.^53^ The remaining 23 fusion genes contained complete promoters and terminators for protein translation (Supplementary Fig. 8a, b, and Supplementary Table 7). Using RT‒PCR and the available tumor samples, we validated *ARID1B-ZDHHC14* and *CSNK1D-CCDC57* (Supplementary Fig. 8c, d), suggesting their potential biological relevance and warranting further investigation.

To further elucidate the intrinsic alterations in SCLC, we analyzed ASE using bulk RNA-seq data and rMATS^54^ (Supplementary Fig. 9a). We identified splicing variants present in tumor but not normal lung tissues. Among the 45 SCLC samples, 18,268 genes exhibited ASEs, of which 3092 were considered significant (Supplementary Table 8). These events included skipped exons (SEs), alternative 5’ splice sites (A5SSs), alternative 3’ splice sites (A3SSs), mutually exclusive exons (MEEs), and retained introns (RIs) (Supplementary Fig. 9b). The most common ASE was SE, which was found in 18,029 (98.7%) of the 18,268 affected genes (Supplementary Fig. 9c, Supplementary Table 8). Among the 45 patients, 1971, 1024, 267, 219, and 409 significant genes were associated with SE, MEE, A3SS, A5SS, and RI, respectively (Supplementary Fig. 9c). In our study, combined SCLC patients (n = 8) presented with ASEs equal to those of SCLC patients (n = 37; Supplementary Table 8).

**Fig. 5:**
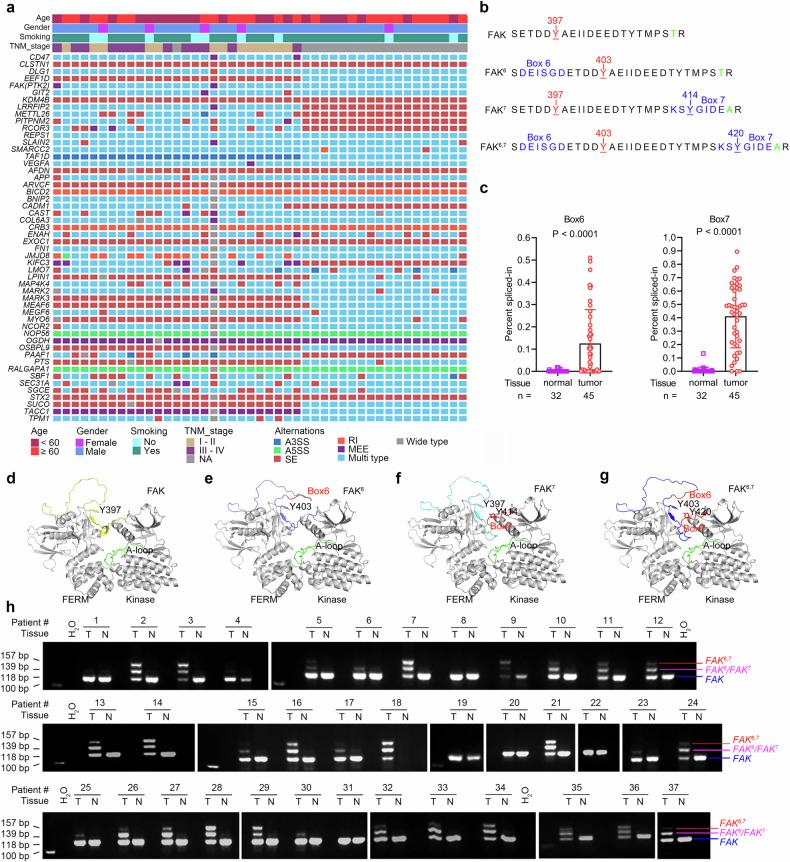
High-frequency alternatively spliced genes in SCLCs. **a** Alternative splicing events (ASEs) in the genes most frequently affected in 45 SCLCs. The tumors are arranged from left to right in the top track. **b** Alignment of FAK, FAK^6^, FAK^7^, and FAK^6,7^. Only the regions flanking Y397 are shown. **c** The percent spliced-in (PSI) values of *FAK* transcripts containing Box 6 and Box 7 in tumor and counterpart normal lung tissues. *P* value, Student’s *t* test. **d-g** Models of the FERM-Kinase region of FAK and its variants. The FERM and kinase domains are colored gray, and the activation (A) loop in the kinase domain is colored green. The variant region is in red for each FAK alternative. The linker between the FERM and kinase domains is in yellow in FAK (**d**), slate in FAK^6^ (**e**), cyan in FAK^7^ (**f**), and blue in FAK^6,7^ (**g**). The Y397 autophosphorylation site in the linker is labeled. The additional tyrosine residues from the insertion (Y414 in FAK^7^ and Y420 in FAK^6,7^) are also labeled. **h** *FAK* variants were detected via RT‒PCR in paired tumor–normal samples from 37 patients, including 27 patients whose samples were analyzed via bulk RNA-seq. Sanger sequencing was used to confirm these results, as shown in Supplementary Fig. 10b, **c**. T tumor tissue, N adjacent normal lung tissue

Sixteen genes, including *Cluster of Differentiation 47* (*CD47*), *Calsyntenin 1* (*CLSTN1*), *Discs Large MAGUK Scaffold Protein 1* (*DLG1*), *Eukaryotic Translation Elongation Factor 1 Delta* (*EEF1D*), and *Focal adhesion kinase* (*FAK*)/*Protein Tyrosine Kinase 2* (*PTK2*)^55^ (Fig. 5a), exhibited ASEs in all 45 patients (Fig. 5a, Supplementary Table 8). Additionally, 36, 18, 25, and 22 genes harbored ASEs in 44, 43, 42, and 41 patients, respectively (Fig. 5a, Supplementary Table 8). A splicing variant of *REST* in which a 50 bp box was inserted between exons 3 and 4 led to the introduction of a frameshift and stop codon and the production of a truncated protein with only 340 amino acids,^56^ which was identified in seven (15.6%) of the 45 SCLC samples (Supplementary Fig. 9d, e). This variant was validated in three tumor samples (Supplementary Fig. 9f) and in two of the four SCLC cell lines (Supplementary Fig. 9g) via RT‒PCR and the corresponding primers (Supplementary Table 16).

### *FAK* splicing variants in SCLCs

#### FAK splicing variants

*FAK* splicing variants were selected for further investigation because these variants are observed at relatively low frequencies in NSCLCs but are not reported in SCLCs.^55^ Moreover, FAK inhibitors have exhibited inhibitory effects on SCLC cells^57^ and induced stable disease in 1/1 patients who received PF562271 in a phase I trial.^58^ In SCLC, two additional spliced boxes of 18 bp (Box 6) and 21 bp (Box 7) were included on either side of the codon encoding autophosphorylation site Y397, designated *FAK*^*6*^, *FAK*^*7*^, and *FAK*^*6,7*^, respectively (Fig. 5b and Supplementary Fig. 10a). In FAK^6^, six amino acids were inserted, and the original Y397 residue became Y403; in FAK^7^ and FAK^6,7^, an additional tyrosine residue, Y414 or Y420, respectively, was inserted after Y397 (Fig. 5b). The Percent-Spliced-In (PSI) values of *FAK* transcripts containing Box 6 and Box 7 in tumor tissues were much greater than those in normal lung tissues (Fig. 5c). In this cohort, the sample size for combined SCLC patients was small (n = 8), and combined SCLC patients seemed to have equal FAK^6/7^ levels as SCLC patients did (n = 37; Supplementary Fig. 9h). However, when bulk RNA-seq data from other cohorts^6^ were included, we found that more FAK splicing variants were detected in pure SCLCs (n = 135) than in combined SCLCs (n = 17; Supplementary Fig. 9i).

#### Structural insights into FAK variants

To assess the potential effects of these ASEs on FAK function, AlphaFold2 was used to predict FAK structure. The wild-type (WT) FAK structure (downloaded from https://alphafold.ebi.ac.uk/) showed high confidence in the FERM, kinase, and FAT domains. Compared with their corresponding crystal structures, the Cα root mean square deviation (RMSD) values for the FERM and kinase domains were 1.046 Å and 0.983 Å, respectively. The predicted structure of FAK adopted an autoinhibited conformation (Fig. 5d), which is consistent with a previous report.^59^ In this conformation, Y397, located at the linker region and sandwiched between FERM and kinase domains, was located away from the activation loop (A-loop) of the kinase domain (Fig. 5d). In FAK^6^, the insertion of Box 6 occurred at the N-terminus of Y403, which was distant from the kinase domain, suggesting that its effect on autophosphorylation may be minimal (Fig. 5e). In FAK^7^ and FAK^6,7^, Box 7 insertion increased the length of the loop at the C-terminus of Y397, allowing greater conformational freedom and potentially facilitating autophosphorylation of Y397 in FAK^7^ and Y403 in FAK^6,7^ (Fig. 5f, g). Furthermore, the additional tyrosine residues in FAK^7^ and FAK^6,7^ may serve as additional phosphorylation sites (Fig. 5f, g), contributing to increased kinase activity and potentially important roles in SCLC carcinogenesis.

#### Validation and visualization of FAK variants in SCLCs

We validated *FAK* variants in additional 109 SCLC samples (Supplementary Table 9). Using RT‒PCR and subsequent Sanger sequencing of 37 tumor-normal paired patient tissues (Supplementary Table 9), we found that in normal lung tissues, only WT *FAK* (*FAK*) was detected (Fig. 5h). Among the 37 tumor samples, 6 (16.2%) displayed only *FAK* bands, 4 (10.8%) presented two bands (*FAK* and *FAK*^*6*^ or *FAK*^*7*^), and 27 (73%) presented three bands (*FAK*, *FAK*^*6*^*/FAK*^*7*^, and *FAK*^*6,7*^) (Fig. 5h). Sanger sequencing of the PCR products confirmed the sequences corresponding to the WT *FAK* and *FAK*^*6,7*^ isoforms (Supplementary Fig. 10b). The middle bands of all patients were cloned and inserted into the pCE2-TA/Blunt-Zero plasmid and sequenced, and both boxes 6 and 7 were detected (Supplementary Fig. 10c, Supplementary Table 10 and 11). Therefore, the patients were classified into three subgroups: *FAK*; *FAK*, *FAK*^*6*^ and *FAK*^*7*^ (hereafter, *FAK*^*6*^/*FAK*^*7*^); and *FAK, FAK*^*6*^/*FAK*^*7*^ and *FAK*^*6,7*^ (hereafter, *FAK*^*6,7*^).

**Fig. 6:**
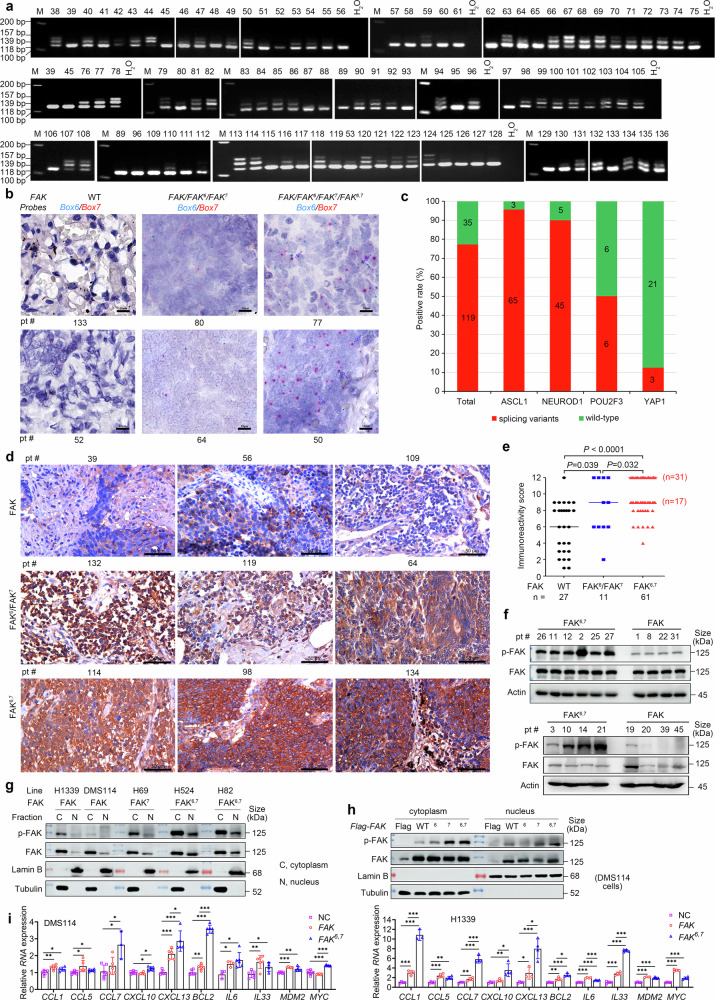
FAK splicing variants in an additional cohort of 99 SCLCs. **a** *FAK* variants in the tumor tissues of patients detected by RT‒PCR. **b** Representative results of *FAK* variants in tumor tissues detected by in situ hybridization BaseScope Duplex assays. **c** Positive rates of *FAK* splicing alternatives across different subtypes of SCLCs. The numbers in the columns indicate positive cases. **d** Representative results of p-FAK in patients with variants of *FAK* in tumor tissues detected by IHC assays. **e** Immunoreactivity scores from the immunohistochemistry assays in patients with *FAK* splicing variants. *P* values, Student’s *t* test. **f** The expression level of p-FAK in patients was detected by Western blotting. **g** The indicated SCLC cells were lysed, and cytosolic and nuclear proteins were separated and subjected to western blotting with the indicated antibodies. **h** DMS114 cells were transfected with the indicated *FAK* transcripts and lysed, and cytosolic and nuclear proteins were isolated for western blotting with the indicated antibodies. **i** DMS114 and H1339 cells were transfected with the indicated *FAK* transcripts and lysed, and RNA samples were isolated for quantitative RT‒PCR

In normal control tissues, no alternative splicing of *FAK* was detected (Fig. 5h and Supplementary Fig. 10b). This prompted further investigation of *FAK* splicing variants in formalin-fixed paraffin-embedded (FFPE) samples (n = 99) via RT‒PCR analysis. We found that 27 (27.3%), 10 (10.1%), and 62 (63.6%) patients harbored *FAK*, *FAK*^*6*^/*FAK*^*7*^, and *FAK*^*6,7*^,, respectively (Fig. 6a, Supplementary Tables 10, 11).

To visualize the single RNA molecules of *FAK* splice variants, BaseScope duplex assays were performed on 56 FFPE samples that had been tested by RT‒PCR using probes targeting Box 6 and Box 7. In 13 samples positive for *FAK* by RT‒PCR, no Box 6 or Box 7 signals were detected; in 5 samples with *FAK*^*6*^/*FAK*^*7*^, both Box 6 (blue) and Box 7 (red) signals were detected; and in 35 samples with *FAK*^*6,7*^, both Box 6 (blue) and Box 7 (red) signals, as well as *FAK*^*6,7*^ (overlapping signals), were detected (Fig. 6b, Supplementary Table 11).

#### FAK splicing variants in ASCL1^+^ and NEUROD1^+^ SCLCs

To determine whether FAK ASE is enriched in specific SCLC subtypes or not, we analyzed the potential associations between *FAK* splicing variants and the four potential subtypes of SCLC. *FAK* variants were present in 65 (95.6%) of 68 *ASCL1*^+^ SCLCs, 45 (90%) of 50 *NEUROD1*^+^ SCLCs, 6 (50%) of 12 *POU2F3*^+^ SCLCs, and 3 (12.5%) of 24 *YAP*^+^ SCLCs (Fig. 6c, Supplementary Table 12), indicating that FAK splicing variants were associated mainly with the *ASCL1*^+^ and *NEUROD1*^+^ SCLC subtypes.

#### Elevated phosphorylation of FAK in patients with splicing variants

We assessed phosphorylated FAK (p-FAK) expression by immunohistochemistry (IHC) in FFPE samples using an antibody against phosphorylated Y397 (in FAK and FAK^7^) or Y403 (in FAK^6^ and FAK^6,7^). The results revealed that patients with FAK^6^/FAK^7^ presented higher p-FAK levels than those with FAK did, whereas patients with FAK^6,7^ presented the highest p-FAK staining level (Fig. 6d). Consistently, patients with FAK^6,7^ had the highest immunoreactivity score (IRS; median, 12), followed by patients with FAK^6^/FAK^7^ (median, 9) and those with FAK alone (median, 6; Fig. 6e). In these patients, p-FAK was localized mainly in the cytoplasm, although nuclear p-FAK was also observed, particularly in patients with FAK^6,7^ and FAK^6^/FAK^7^ (Fig. 6d). Western blot analysis confirmed that patients with FAK^6,7^ expression had higher p-FAK expression than those with FAK alone (Fig. 6f).

To confirm the increased nuclear p-FAK expression in patients with FAK splicing variants, a subcellular fractionation assay was performed in SCLC lines. We found that FAK^6,7^-expressing H524 and H82 lines presented increased nuclear p-FAK expression compared with FAK-expressing H1339 and DMS114 cells (Fig. 6g). Moreover, increased nuclear p-FAK expression was detected in DMS114 cells transiently transfected with *FAK*^*7*^ and *FAK*^*6,7*^ compared with that in cells transfected with *FAK* (Fig. 6h). Given that nuclear FAK is able to modulate the expression of the chemokine genes *C-C motif chemokine ligand 5* (*CCL5*), *CCL10*, *CCL1*, *CCL7* and *CXCL13*,^60^ *interleukin 6* (*IL6*),^61^ *IL33*,^62^ the apoptosis regulator *BCL2*,^63^ and the proto-oncogene *MYC* and *MDM2*,^64^ we tested the potential effects of FAK variants on these genes in WT *FAK*-bearing DMS114 and H1339 cells that were transfected with *FAK* or *FAK*^*6,7*^ transcripts. Using qPCR, we showed that both FAK and FAK^6,7^ upregulated these genes; in these cells, the effects of FAK^6,7^ on *CCL7*, *CXCL13*, and *BCL2* were significantly greater than those of FAK (Fig. 6i). However, whether these changes in mRNA expression result in increased protein production remains to be determined by ELISA or western blot.

#### FAK splicing variants in SCLC cell lines

We analyzed the expression of *FAK* splicing variants in SCLC cell lines using data from the CCLE dataset on the Cancer Dependency Portal (DepMap; https://depmap.org/portal/ccle/)^65^ and found that 40 (87.0%) of the 46 SCLC cell lines investigated harbored Box 6/Box 7 (Supplementary Fig. 11a). In RT‒PCR and sequencing analyses of 10 cell lines, only WT *FAK* was detected in normal lung epithelial 16HBE cells (Supplementary Fig. 11b). Among the nine lines, two (22.2%; DMS114 and H1339) expressed *FAK*, 6 (66.7%; H82, H446, H524, H1688, H2227, and DMS153) expressed *FAK*^*6,7*^ and one (11.1%; H69) expressed the *FAK*^*7*^ variant (Supplementary Fig. 11b). Western blot assays revealed that p-FAK levels were low in H1339 and DMS114 cells but high in H69, H82, H446, H524, and DMS153 cells (Supplementary Fig. 11c).

#### Increased tyrosine kinase activity of FAK splicing variants

To evaluate the functions of the alternative splicing proteins, plasmids containing *FAK* variants were transfected into DMS114 cells, which were subsequently lysed 48 h later for Western blotting. We found that in cells transfected with *FAK*^*7*^ and *FAK*^*6,7*^, the expression levels of p-FAK (Y397), p-FAK (Y576/577), and p-FAK (Y925) were greater than those in cells transfected with *FAK* and *FAK*^*6*^ (Supplementary Fig. 11d). In cells transfected with *FAK*^*7*^ and *FAK*^*6,7*^, p-mTOR and p-AKT levels were elevated (Supplementary Fig. 11d). FAK proteins were purified from the cells, and tyrosine kinase activity was evaluated.^55^ We found that FAK^7^ and FAK^6,7^ exhibited significantly greater kinase activities than FAK did (Supplementary Fig. 11e). In addition, cotransfection with *FAK*^*6,7*^ and *FAK*^*7*^ substantially increased p-FAK levels compared with those in cells transfected with *FAK*^*6*^ alone, and cotransfection with *FAK*^*6,7*^ markedly increased p-FAK levels in cells transfected with either *FAK*^*6*^ or *FAK*^*7*^ alone (Supplementary Fig. 11f).

To determine the role of mTOR/AKT signaling in FAK dependence in certain cell lines, we treated SCLC cell lines with the AKT inhibitor MK-2206 and the mTOR inhibitor Torin 1 and evaluated their impacts on cell proliferation. The results revealed that MK-2206 (2 μM) moderately suppressed, whereas Torin 1 (0.2 μM) markedly inhibited, the growth of FAK-harboring DMS114 and SBC-5 cells (Supplementary Fig. 11g). However, FAK^7^/FAK^6,7^-expressing cells presented reduced sensitivity to the mTOR inhibitor (Supplementary Fig. 11g), suggesting that SCLC cells with FAK variants may survive mTOR inhibition, possibly through increased activity of the AKT and Bcl-2/Myc pathways.

#### FAK splicing alternatives are associated with poor prognosis in SCLCs

**Fig. 7:**
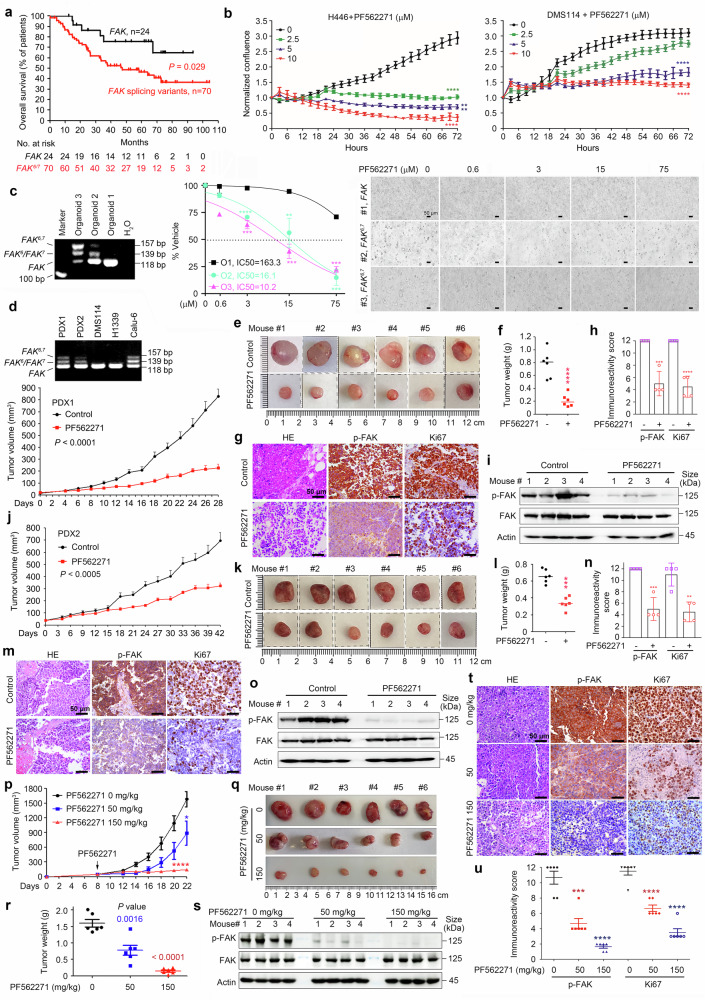
Clinical significance of *FAK* splicing variants in SCLC. **a** OS of patients with SCLC with wild-type *FAK* and those with splicing variants. *P* value, log-rank test. **b** H446 and DMS114 cells were treated with 2.5 to 10 μM PF562271 and monitored with an IncuCyte live-cell analysis system. *P* values, Student’s *t* test. ****, *P* < 0.0001. **c** Three SCLC patient-derived organoids were established and treated with PF562271, and organoid cell viability was quantified via the CCK-8 assay. Representative images are shown. O, organoid. *P* value, Student’s *t* test. **, *P* < 0.01; ***, *P* < 0.001; ****, *P* < 0.0001. **d** *FAK* in the two patient-derived xenograft (PDX) models was detected by RT‒PCR (upper panel). The PDX1 mice were treated with PF562271 at 50 mg·kg^−1^·day^−1^ 5 days per week for four weeks. The tumor volume was estimated every 2 days. The data are shown as mean ± sd. N = 6 for each group. *P* value, Student’s *t* test. **e** Images of xenograft tumors isola*t*ed from the mice. **f** Weights of xenograft tumors isolated from the mice. *P* value, Student’s *t* test. ****, *P* < 0.0001. **g, h** Representa*t*ive images of hematoxylin‒eosin (HE) staining and IHC assays for p-FAK and Ki67 in tumor sections harvested from PF562271-treated and vehicle control-treated mice (n = 4 for each group; **g**). The immunoreactivity scores of p-FAK and Ki67 were calculated (**h**). *P* values, two-tailed unpaired *t* test. ***, *P* < 0.001; ****, *P* < 0.0001. **i** Western blot assays using lysates of tumor samples harvested from four mice in each group. **j** Mice bearing PDX2 tumors were treated with 50 mg·kg^−1^·day^−1^ PF562271, and the tumor volume was estimated every 3 days. The data are shown as mean ± sd. N = 6 for each group. *P* value, Student’s *t* test. **k** Images of xenograft tumors isolated from the mice. **l** Weights of xenograft tumors isolated from the mice. *P* value, Student’s *t* test. ***, *P* < 0.001. **m, n** Representative images of HE staining and IHC assays of p-FAK and Ki67 in tumor sections harvested from PF562271- and vehicle control-treated mice (n = 4 for each group; **m**). The immunoreactivity scores of p-FAK and Ki67 were calculated (**n**). *P* values, two-tailed unpaired *t* test. **, *P* < 0.01; ***, *P* < 0.001. **o** Western blot assays using lysates of tumor samples harvested from the mice. **p** H82 cell-inoculated mice were treated with PF562271 at the indicated dosage, and the tumor volume was estimated every two days. The data are shown as mean ± sd. N = 6 for each group. *P* value, Student’s *t* test. *, *P* < 0.05; ****, *P* < 0.0001. **q** Images of xenograft tumors isolated from the mice. **r** Weights of xenograft tumors isolated from the mice. *P* value, Student’s *t* test. **s** Western blot assays using lysates of tumor samples harvested from the mice. **t, u** Representative images of HE staining and IHC assays of p-FAK and Ki67 in tumor sections (n = 6 for each group; **t**). The immunoreactivity scores of p-FAK and Ki67 were calculated via the IHC assay results (**u**). *P* values, two-tailed unpaired *t* test. ***, *P* < 0.001; ****, *P* < 0.0001

We analyzed the potential associations between *FAK* variants and the prognosis of patients whose survival information was available, including 24 patients with *FAK*, 10 with *FAK*^*6*^/*FAK*^*7*^, and 60 with *FAK*^*6,7*^. We found that, compared with those with *FAK splicing variants*, patients with SCLC and *FAK* splicing variants had a significantly worse prognosis (Fig. 7a, *P* = 0.029), suggesting that alternative FAK splicing variants may play a critical role in SCLC pathogenesis.

#### SCLC cells with splicing variants are sensitive to FAK inhibitors

We tested the effects of siRNA-mediated silencing of *FAK* on cell proliferation and found that inhibition of FAK slightly repressed DMS114 and significantly inhibited H446 proliferation, as revealed by the CCK8 assay-based inhibition rate of proliferation (Supplementary Fig. 12a). We tested the effects of FAK inhibitors on SCLC cells and found that PF562271^58^ at 0.25 to 1.5 μM had much lower inhibitory effects on FAK-expressing lines than on FAK^6,7^-harboring lines (Supplementary Fig. 12b). PF562271 at 2 μM was unable to inhibit H1339 cell growth but significantly repressed the growth of H446 cells (Supplementary Fig. 12c). PF562271 at 1 to 5 μM markedly inhibited the proliferation of H82 and H524 cells (Supplementary Fig. 12d).

PF562271 arrested the cell cycle at the G2/M phase in H446 and H524 cells (Supplementary Fig. 12e). Another FAK inhibitor, PF573228, also arrested cell cycle at the G2/M phase in the cells (Supplementary Fig. 12f). Both compounds moderately inhibited the colony-forming activity of DMS114 cells and significantly suppressed that of H446 cells (Supplementary Fig. 12g, h). FAK inhibitors also reduced H446 and DMS114 cell migration (Supplementary Fig. 12i, j). Using western blot assays, we showed that FAK inhibitors decreased p-FAK expression, particularly in cells with *FAK*^*6,7*^ (Supplementary Fig. 12k, l).

Because these cell lines have been established for decades and have obvious limitations, we tested the effects of PF562271 on patient-derived organoid (PDO) and patient-derived xenograft (PDX) models. Three SCLC PDOs were established using randomly obtained samples as described previously,^66^ among which two harbored *FAK*^*6,7*^ (Fig. 7c). We found that while PF562271 slightly inhibited the growth of *FAK* PDOs, it drastically suppressed the growth of *FAK*^*6,7*^ PDOs (Fig. 7c). We tested the effects of PF562271 on *FAK*^*6,7*^-harboring PDX models (Fig. 7d). In PDX model 1, which was derived from a male patient with *FAK*^*6,7*^ and mutations in *TP53* and *Rb1*, PF562271 at a relatively low dose (50 mg/kg) substantially inhibited tumor growth by 73.5% (Fig. 7d, e) and reduced tumor weight by 75% (Fig. 7f). IHC analysis revealed that PF562271 markedly downregulated the expression of p-FAK and Ki67 (Fig. 7g, h). Downregulation of p-FAK in PF562271-treated mice was confirmed by western blot analysis of tumor sample lysates (Fig. 7i). In PDX Model 2, which was derived from a female patient with *FAK*^*6,7*^ and mutant *TP53* and *Rb1*, PF562271 strongly inhibited tumor growth (Fig. 7j, k), reduced tumor weight (Fig. 7l), and suppressed the expression of p-FAK and Ki67 (Fig. 7m–o). In addition, PF562271 at 50 mg/kg/day inhibited tumor growth in the H82-xenograft murine model and effectively suppressed tumor growth at 150 mg/kg/day, with substantial reductions in p-FAK and Ki67 levels (Fig. 7p–u).

### Smoking status and tumor stage do not affect microbiota variance in SCLC

To investigate the potential role of the microbiota in SCLC, 16S rRNA gene sequencing was performed on tumor-adjacent normal lung-paired samples harvested from 53 patients (Supplementary Table 1). There was no significant difference in the proportion of bacterial DNA between tumor and normal lung tissues (Supplementary Fig. 13a) or in the microbiota alpha diversity indices (Shannon index and observed genera, Supplementary Fig. 13b, c) or overall microbiota composition (Supplementary Fig. 13d). In both tumor and normal tissues, Proteobacteria, Firmicutes, and Bacteroidetes were the dominant phyla (Supplementary Fig. 13e), with *Lactobacillus*, *Acinetobacter* and *Methylobacterium* being the dominant genera (Supplementary Fig. 13f). In addition, the variance in the microbiota of the tumor tissue was not associated with tumor stage (*R*^*2*^ = 5.78%, *P* = 0.46, PERMANOVA) or smoking status (*R*^*2*^ = 2.34%, *P* = 0.21).

### Eleven high-frequency mutations are identified in addition to *RB1* and *TP53*

We conducted WES to analyze somatic exonic mutations in 111 SCLC samples (Supplementary Table 1). The cohort presented a mutation rate of 5.88 mutations per million base pairs (Mb) and a median nonsynonymous mutation rate of 4.74 mutations per Mb. Smokers had a higher nonsynonymous mutation rate than nonsmokers did (6.08 vs 3.44 mutations per Mb, *P* = 0.006; Supplementary Table 13). In addition, “hot” tumors presented higher tumor mutation burden (TMB) than “cold” tumors did (8.00 vs 4.32 mutations per Mb; *P* = 0.07, Supplementary Fig. 7f).

In this cohort, 4782 nonrecurrently and 7425 recurrently (mutated in two or more patients) mutated genes were identified (Supplementary Table 14). Among the recurrently mutated genes, 4805 had a ratio of nonsynonymous to synonymous mutations (dN/dS) > 2. These genes fell into 533 categories, with homophilic cell adhesion via plasma membrane adhesion molecules (GO) and ABC transporters (KEGG) being the most frequently mutated genes (Supplementary Fig. 14a, b). Calcium signaling and ion channel genes had relatively high mutation rates in SCLCs (Supplementary Fig. 15), similar to those in NSCLCs with long-term exposure to indoor air pollution.^67,68^

**Fig. 8:**
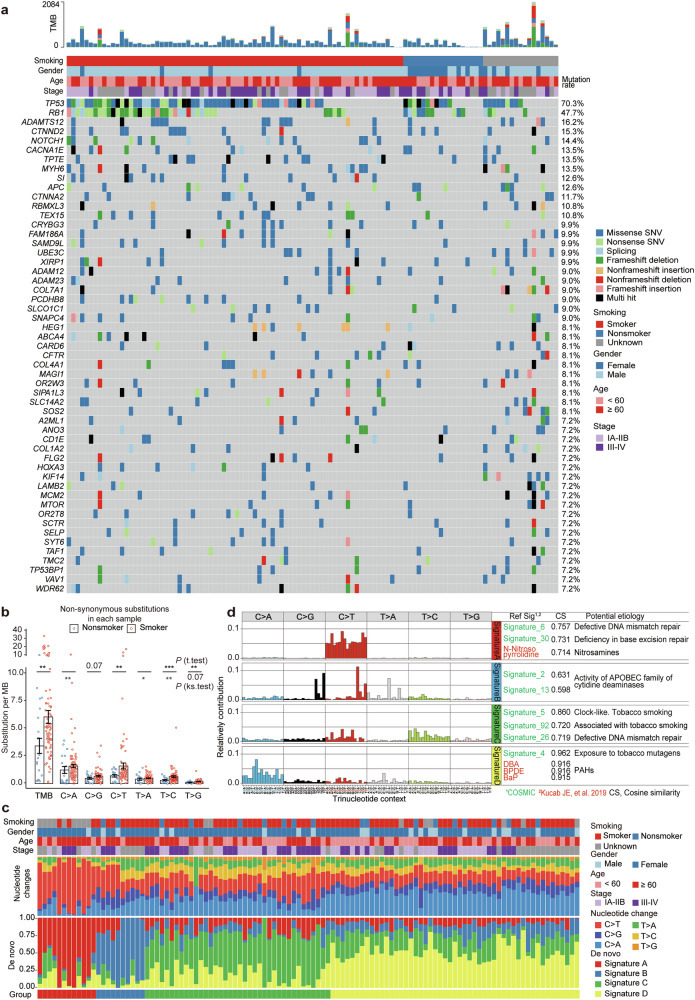
Somatic exonic mutations and carcinogen signatures in SCLC. **a** Somatic exonic mutations in SCLCs. Tumors are arranged from left to right in the top track, alterations in candidate genes are annotated for each sample according to the color panel, and the mutation rates for each gene are shown in the right panel. **b** Nucleotide substitutions in smokers and nonsmokers. TMB, tumor mutation burden. *P* values, two-sided Student’s *t* test. *, *P* < 0.05; **, *P* < 0.01; ***, *P* < 0.001. **c** Nucleotide changes in each patient. The tumors are arranged from left to right in the top track, the demographic characteristics of the patients are annotated according to the right-side color panel, and the de novo signatures A, B, C, and D are described in (**d**). **d** De novo signatures A through D. COSMIC and carcinogen signatures^75^ are used as references, and potential etiologies are shown

*TP53* and *RB1* were mutated in 78 (70.3%) and 53 (47.7%) of the 111 patients, respectively (Fig. 8a, Supplementary Fig. 16). Our WES and bulk RNA-seq data revealed that, in *Rb1* WT tumors (n = 18), the expression of *Cyclin D1*, *D2* and *D3* was slightly (but not significantly) greater than that in *Rb1* mutant tumors (n = 26), which is consistent with the results of George et al.^2^ and Jiang et al.^5^ (Supplementary Fig. 17a). No significant differences in *Cyclin D1*, *D2* or *D3* expression were observed between *TP53* WT (n = 10) and *TP53* mutant tumors (n = 34), which is consistent with the results of George et al.^2^ and Jiang et al.^5^ (Supplementary Fig. 17b). These results suggest that there may be other alterations in the p53 and Rb1 pathways in SCLC that warrant further investigation.

We identified 11 other significant genes (*P* values < 0.05) with mutation rates >10%, including *ADAMTS12* (16.2%), *CTNND2* (15.3%), *NOTCH1* (14.4%), *CACAN1E* (13.5%), *TPTE* (13.5%), *MYH6* (13.5%), *SI* (12.6%), *APC* (12.6%), *CTNNA2* (11.7%), *RBMXL3* (10.8%), and *TEX15* (10.8%) (Fig. 8a and Supplementary Fig. 16). There were 404 significant genes with mutation rates between 5% and 10% (Supplementary Table 14). Compared with patients of Caucasian ancestry with SCLC,^2–4^ Chinese patients presented a slightly lower *TP53* mutation rate (Fisher’s exact test, *P* = 0.2), a significantly lower *RB1* mutation rate (Fisher’s exact test, *P* = 0.05; Supplementary Fig. 18), and significantly higher mutation rates in 17 genes (Supplementary Fig. 18).

### Mutational signatures of environmental carcinogens

Analysis of nucleotide changes revealed that C:G→A:T transversions and C:G→T:A transitions were the most abundant nucleotide substitutions, with smokers exhibiting higher rates than nonsmokers (Supplementary Table 15, Fig. 8b). In smokers, the mean number of C:G→T:A transitions (6.71 per Mb) was greater than the mean number of C:G→A:T transversions (5.95 per Mb; Supplementary Table 15). Furthermore, the mean C:G→G:C transversions (a signature of the tobacco carcinogen 1,3-butadiene^69^) and mean T:A→A:T transversions (a signature of the air pollutant vinyl chloride^70^) were also high in these patients (Supplementary Table 15, Fig. 8b, c). The T:A→C:G transition, which is usually induced by the tobacco carcinogen N-nitrosodiethylamine^71^ and a widely used synthetic industrial chemical, 1,4-dioxane,^72^ resulted in 3.11 mutations/Mb in smokers and 1.83 mutations/Mb in nonsmokers (*P* = 0.04; Supplementary Table 15). We analyzed the nucleotide substitutions within the 5’ and/or 3’ sequence context of each mutated base^22^ in each patient and found that signature 4, characteristic of PAH exposure,^22^ was the most frequently observed signature in both smokers and nonsmokers (Supplementary Fig. 19, Supplementary Table 15). Signatures 5, 24, 39, and 87 were also relatively high in these patients (Supplementary Table 15).

Using the nonnegative matrix factorization algorithm^73^ and mutational patterns,^74^ we obtained four de novo mutational signatures A, B, C, and D (Fig. 8d), that showed similarity to the signatures associated with various intrinsic or extrinsic factors (Supplementary Fig. 20a). Signature A comprises predominant C>T mutations and very few C>A transversions, indicating potential exposure to NNK^23^ and N-Nitrosopyrrolidine^75^ and defective DNA mismatch repair.^22^ Signature B was characterized by mutations distributed across all 96 subtypes of base substitutions, with relatively high levels of C>T, C>G, and T>A mutations. Signature C exhibited a relatively high proportion of C>T and T>C mutations, whereas Signature D was characterized by C>A substitutions alongside the other 5 mutation types (Fig. 8d). The 111 patients were clustered into four groups according to these de novo signatures: group 1 (12 patients, 10.8%) was characterized by Signature A, group 2 (10 patients, 9%) was characterized by Signature B, group 3 (38 patients, 32.2%) was characterized by Signature C, and group 4 (51 patients, 45.9%) was characterized by Signature D (Fig. 8c).

We analyzed the nucleotide substitutions according to mutational signatures of selected environmental agents^75^ and evidence of exposure to tobacco-related compounds such as benzo[a]pyrene-7,8-dihydrodiol-9,10-epoxide (BPDE)/BaP, dibenz[a,j]acridine (DBAC), methyleugenol, dibenz[a,h]anthracene (DBA), and formaldehyde in the genomes of these patients (Supplementary Fig. 20a, Supplementary Table 15). Analysis of mutational spectra and their proportions of genomic somatic mutations revealed similarities in environmental signatures between smokers and nonsmokers (Supplementary Fig. 20b), suggesting potential exposure to secondhand smoke and air pollutants in nonsmokers. In addition, combined SCLC patients (n = 12) presented exonic alterations similar to those of SCLC patients (n = 99; Supplementary Table 15).

## Discussion

At least two key questions remain to be addressed in our ongoing efforts to tame SCLC, one of the most lethal malignancies worldwide. First, while high-frequency loss-of-function mutations in tumor suppressor genes have been identified, it remains essential to identify high-frequency gain-of-function alterations in oncogenes that play a crucial role in SCLC pathogenesis. Second, suitable targets, e.g., constitutively activated tyrosine kinases resembling the epidermal growth factor receptor (EGFR) in NSCLC, should be identified for the development of targeted therapies. To address these questions and further elucidate ITH, we collected tumor samples from 314 patients with SCLC and conducted multiomics studies.

High-frequency mutations in oncogenes and tumor suppressor genes play critical roles in tumorigenesis. High-frequency loss-of-function mutations have been detected in tumor suppressors, including *TP53*, *Rb1*, and *APC*. However, high-frequency (e.g., >10%) gain-of-function mutations in oncogenes have not been identified thus far. Therefore, we analyzed the splicing alternatives in which splice sites were differentially utilized to activate target genes. Interestingly, 18,268 (82%) of the 22,287 human genes^76^ exhibited ASEs in our SCLC cohorts that underwent bulk RNA-seq. ASE represents an emerging feature of SCLC; 16 genes had ASEs in all 45 patients, and 36, 18, 25, and 22 genes harbored ASEs in 44, 43, 42, and 41 patients, respectively. We validated the ASEs of two selected genes, *REST* and *FAK*, via RT‒PCR and Sanger sequencing. These findings underscore the importance of alternative splicing in SCLC and the accuracy of the tool rMATS.^54^ We showed that *FAK* splicing alternatives were present in 119 (77.3%) of the 154 SCLCs, mainly in the SCLC-A, SCLC-N, and SCLC-P subtypes. Structural analysis suggested that FAK^7^/FAK^6,7^ were gain-of-function alterations in that the elongated loop at the C-terminus facilitates Y397/Y403 autophosphorylation, and the additional Y414/Y420 provides more autophosphorylation potential. Our functional studies confirmed that FAK^7^/FAK^6,7^ had elevated tyrosine kinase activity; increased FAK phosphorylation at Y397, Y576/577, and Y925; and increased p-mTOR, p-AKT, and p-p70S6K expression levels compared with those of FAK. Compared with FAK, FAK^6,7^ showed increased transcriptional regulatory activity. Furthermore, FAK splicing variants were associated with a worse prognosis. Hence, alternative FAK splicing represents a high-frequency gain-of-function alteration in oncogene in SCLC. Moreover, 34 of 45 (75.6%) pancreatic neuroendocrine neoplasms and 14 of 15 (93.3%) breast neuroendocrine carcinomas were positive for *FAK*^*6/7*^, ^77^ suggesting that *FAK*^*6/7*^ could be potential biomarkers for neuroendocrine neoplasms.

SCLC has recently been classified by the expression of *ASCL1*, *NEUROD1*, and *POU2F3*, but *YAP1* does not exclusively define a subtype in scRNA-seq studies with small sample sizes.^9^ We found that among the tumor samples from 39 patients, one patient was classified as SCLC-Y, suggesting the need to further dissect SCLC subtypes in more patients. Moreover, using scRNA-seq data containing 432,959 single cells, including 190,313 cancer cells (to our knowledge, the largest cohort thus far), we showed that SCLC-A-*MKI67* and SCLC-A-*CRIP2* represented the two dominant cell clusters in SCLC. While the SCLC-A-*MKI67* cluster could be eliminated by initial treatment, the SCLC-A-*CRIP2* cluster remained the major cancer cell type in the posttreatment samples, suggesting its potential role in drug resistance and relapse. In addition, SCLC ITH was further complicated by heterogeneous expression of MHC-I in SCLC cell populations, as HLA-A, HLA-B, and HLA-C were low in six clusters and high in five clusters according to our scRNA-seq data, and the observation that SCLC cancer cells express MHC-I molecules was confirmed by our spatial proteomics data. These data deepen our understanding of SCLC ITH and cancer cell biology.

Previous studies have indicated that SCLC patients exhibit greater immune sequestration and less immune infiltration than LUAD patients do.^9^ Here, the immunosuppressive microenvironment of SCLC was confirmed, but the differences in immune infiltration between SCLC and NSCLC were not significant. We found that B and T/NK cells were suppressed in tumor tissues but upregulated following neoadjuvant treatment, particularly pB-*IGHA2* and CD8_Tem-*GZMK* cells, which were more prevalent in patients who achieved CR/PR than in those with SD/PD, suggesting their antiSCLC activity. The reduction in B cells in extensive SCLC may be attributed to the significant reduction in TLSs, where mature TLSs normally serve as critical hubs for B cell clonal selection, activation, and differentiation into tumor antigen-targeting plasma cells through somatic hypermutation and antibody class switching.^78^ Additionally, the immunosuppressive TME of extensive-stage SCLC also suppresses B cell infiltration. MHC-I and MHC-II molecules are expressed at low levels in cancer cells but can be upregulated by neoadjuvant therapy, but the underlying mechanisms remain incompletely understood. Hence, further investigations are still needed to elucidate the mechanisms of the immunosuppressive SCLC TME and strategies to improve the efficacy of immunotherapy.

SCLC is thought to originate predominantly from pulmonary neuroendocrine cell and less frequently from surfactant protein C-positive AT2 cell with *TP53* and *Rb1* loss.^79^ SCLC can also arise from LUAD via histological transformation through a stem-like intermediate that closely resembles a pulmonary basal cell.^80^ Indeed, a *PLCG2*-high SLC population was found to recur across SCLC subtypes and is associated with worse overall survival.^9^ We identified an SLC cluster that was present across subtypes. These SLCs expressed high levels of stem cell markers. Given the sample size, further studies with larger cohorts are needed to evaluate the association between SLC and female/nonsmoking status. Similarly, treatment (especially chemotherapy) can eliminate sensitive subpopulations, allowing SLCs to prevail and drive disease progression.^12^ Whether chemotherapy enriches SLCs and promotes SCLC relapse needs to be determined. In addition, tobacco and carcinogenic compounds can induce loss-of-function mutations in *TP53*,^21^ activate Myc and Bcl-2,^81^ and upregulate Sox2, CD133 and CD166 expression.^82–84^ Hence, the role of these carcinogens in promoting stemness and SCLC initiation remains to be further investigated.

SCLC is strongly associated with exposure to smohaze,^13,85^ but no carcinogenic compound has been found to be able to induce SCLC in animal studies. Using WES, we found that SCLC genomes harbor many mutations that represent signatures of environmental carcinogens, such as NNK, PAHs, 1,3-butadiene, 1,4-dioxane, vinyl chloride, and N-nitrosodiethylamine. However, there was no significant difference in the microbiota composition between tumor and normal lung tissues. Furthermore, nonsmoking patients presented environmental signatures similar to those of smoking patients, indicating their potential heavy exposure to secondhand smoke and air pollutants. These findings highlight the need for further exposome studies to dissect SCLC tumorigenesis. Animal models more closely resembling human lung physiology, such as ferret,^86^ should be used to identify the carcinogenic compounds that cause SCLC in humans.

FAK is an oncoprotein critical for cancer cell proliferation, survival, invasion, metastasis, stem cell activity, and immune evasion.^60,87^ It is overexpressed in various malignancies and is associated with poor clinical outcomes.^87–90^ Preclinical studies have shown the anticancer efficacy of FAK inhibition,^91^ and six groups of FAK inhibitors have been developed: inhibitors of ATP binding sites (ATP competitive inhibitors), which block FAK phosphorylation and exhibit good preclinical antiproliferative action against different solid tumors; inhibitors of the FAK-FERM domain, which prevents FAK phosphorylation at Y397; inhibitors of the FAK-FAT domain, which hinder Y965 phosphorylation; allosteric inhibitors (non-ATP-competitive inhibitors), which bind an allosteric site and disrupt specific protein‒protein interactions (e.g., p53-FAK interaction), which results in more selective FAK inhibition; FAK PROTAC degraders; and FAK-based dual-target inhibitors. These inhibitors suppress FAK downstream pathways and inhibit cell survival, proliferation, migration, invasion, and angiogenesis. Eight FAK inhibitors have entered clinical trials; however, only modest clinical activity has been achieved when these drugs are tested as single agents for cancer treatment.^91,92^ Merlin deficiency predicts FAK inhibitor sensitivity,^93^ but more biomarkers for FAK-targeting therapies are still needed. The success of EGFR inhibitors in patients with EGFR mutations^94^ suggests that constitutively activated FAK isoforms may be more sensitive to kinase inhibitors and could serve as biomarkers for FAK inhibitors in cancer patients. This possibility was confirmed by our findings that SCLC cells expressing FAK^7^/FAK^6,7^ were sensitive to inhibitors and that PF562271 significantly inhibited tumor growth in the two PDO and two PDX models and repressed tumor growth in an SCLC cell xenograft murine model. In a phase I trial, one patient with SCLC who received PF562271 achieved SD for six or more cycles.^58^ Therefore, clinical trials testing FAK inhibitors in patients with SCLC should be conducted using splicing variants as biomarkers, and specific inhibitors for constitutively activated FAK^6,7^/FAK^7^ need to be developed for clinical testing.

## Materials and methods

### Patient samples

The institutional review board (IRB) of all participating hospitals approved this study (NCC2020A190), and human tumor and blood samples were obtained from patients under IRB-approved protocols following written informed consent. All tumor samples were reviewed by at least two independent expert pathologists, and the diagnosis of SCLC was histomorphologically confirmed by hematoxylin and eosin (HE) staining and IHC for chromogranin A (CgA), synaptophysin (SYN), CD56 and Ki67. Fresh-frozen tumor and adjacent normal lung tissues and FFPE samples (Supplementary Table 1) were collected from multiple collaborating institutions. Additional information, including the age and sex of the participants, is included in Supplementary Table 1.

### Mice

Nonobese diabetic (NOD)/ShiLtJGpt-Prkdc^em26Cd52^Il2rg^em26Cd22^/Gpt (null; NCG) male mice were purchased from GemPharmatech (San Diego, CA, USA). Four- to six-week-old mice were used for the experiments. All the animal studies were conducted according to protocols approved by the Animal Ethics Committee of our hospital, with the approval ID NCC2019A188. All the mice used in this study were bred and maintained in a specific pathogen-free environment.

### Cells

SCLC cell lines DMS153 and DMS114 (recently suggested to be non-SCLC lines), H69, H82, H446, H524, H2227, H1339, and H1668 (ATCC, Manassas, VA, USA), the normal human lung epithelial cell line 16HBE (Merck Ltd., Beijing, China), and the embryonic kidney HEK293 cell line (ATCC) were cultured in DMEM or RPMI 1640 medium supplemented with 10% fetal bovine serum (Gibco/BRL, Grand Island, NY, USA) at 37 °C in 5% CO_2_. The indicated cells were transfected with plasmids containing *FAK* splicing variants or treated with the FAK inhibitors PF562271 or PF573228 at 0.003 to 10 μM for up to 72 h, and cell proliferation, the cell cycle, and colony formation and migration activities were analyzed.

### Single-cell RNA sequencing

All biopsy procedures were performed following the hospital standard operating procedures, and one to three 20‐gauge tissue core samples were collected; alternatively, 100 mg of tissue was collected after lung cancer surgery. The samples were evaluated by at least two pathologists to confirm the diagnosis. Fresh tissues were stored in sCelLive^TM^ Tissue Preservation Solution (Singleron, Nanjing, Jiangsu, China) and kept on ice for 30 min postsurgery. The samples were washed three times with Hanks’ balanced salt solution (HBSS), minced into small pieces, and digested with three mL of CelLiveTM Tissue Dissociation Solution using the Singleron PythoN™ Tissue Dissociation System at 37 °C for 15 min. The cell suspension was collected and filtered through a 40-micron sterile strainer. GEXSCOPE^®^ red blood cell lysis buffer (Singleron) was added, and the mixture (cell:buffer = 1:2 [volume ratio]) was incubated at room temperature for 5–8 min to remove red blood cells. The mixture was then centrifuged at 300 × *g* at 4 °C for 5 min to remove the supernatant, and the cells were gently resuspended in phosphate-buffered saline (PBS).

Single-cell suspensions (2 × 10^5^ cells/mL) in PBS (HyClone) were loaded onto a microwell chip using the Singleron Matrix^®^ Single Cell Processing System. Barcoding beads were subsequently collected from the microwell chip, followed by reverse transcription of the mRNA captured, cDNA synthesis, and PCR amplification. Amplified cDNA was fragmented and ligated using sequencing adapters. The scRNA-seq libraries were constructed according to the protocol of the GEXSCOPE^®^ Single Cell RNA Library Kits (Singleron).^95^ Individual libraries were diluted to 4 nM, pooled, and sequenced on an Illumina NovaSeq 6000 with 150 bp paired-end reads.

### Cell type determination

For scRNA-seq data analysis, the expression matrix of single cells in each sample was obtained using the CeleScope software (v1.9.0) (https://github.com/singleron-RD/CeleScope). Low-quality samples (RNA counts <600, feature genes <200, and percentage of mitochondrial RNAs >20%) were excluded. The different datasets were integrated using Seurat tools (v4.0), with batch effects removed by canonical correlation analysis (CCA).^96^ Cells were clustered using UMAP after scaling by percentage of mitochondrial RNA, number of gene features per cell, percentage of ribosomes, and RNA counts. Cell types were defined based on the expression of feature genes (e.g., *ASCL1*, *CD3D*, *CD3E*, *CD14*, *CD68*, *CD79A*, *CD79B*, *CLDN5*, *COL1A2*, *EPCAM*, *MRC1*, and *PECAM1*). Initially, these cells were broadly classified as NE/cancer cells, T/NK cells, B cells, monocytes/Mφs, AT1/AT2/basal/ciliated/club cells, endothelial cells, and stromal cells. We performed additional clustering on these broad cell categories to further refine the cell types. Cell types were defined by combining major markers and feature genes. For example, within the T/NK cell population, subtypes are determined on the basis of the expression of genes such as *CD4*, *CD8A*, and *CD8B*, and then, clusters are named according to their feature genes. Similarly, B cell subtypes were identified on the basis of genes such as *CD38*, *IGHG1/2/4*, *MS4A1*, and *CD19* and the cluster assigned by integrating their feature genes. Differentially expressed genes were identified via the FindMarkers or FindAllMarkers algorithm in Seurat. TopGO tools (v 3.13) (http://bioconductor.org/packages/release/bioc/html/topGO.html) in the R package were used for gene ontology analysis. GSEA was performed using the GSEA tools (v4.0.3). For the enrichment of club, basal, ciliated, neuroendocrine, and AT2 cells, feature genes were obtained from cell subtype-specific genes identified in previous single-cell transcriptomic studies of normal lung tissue.^97,98^ Cancer cells were distinguished based on their epithelial origins, clustering patterns, and large-scale chromosomal CNVs using the inferCNV tool (https://github.com/broadinstitute/inferCNV; v1.2.1).^9,11^ For inferCNV analysis, 20% of the cells were randomly sampled, and AT1/AT2/Basal/Ciliated/Club cells were used as references to analyze the CNV characteristics of the tumor cells, with the samples being sorted accordingly.

We used CytoTRACE2 to analyze the pluripotency of single cells. We randomly selected 20% of the SCLC and AT2/basal/ciliated cells for calculation, using the count slot parameter, and the results are shown as a CytoTRACE2 boxplot according to phenotype. Cell‒cell interaction analysis was performed using CellChat, which calculates individual interactions between different cell subpopulations in each sample. Subpopulations with fewer than 10 cells were excluded from the interaction analysis. We extracted and analyzed interactions related to the MHC-I and MHC-II pathways for display. For pathway enrichment, we used the scMsigdbScoring and scScoreDimPlot packages in Yeskit.^99^ We used the Gene Ontology Biological Process dataset from the GSEA database, which includes pathways such as APP via MHC class I and class II, for MHC pathway enrichment analysis.

### Whole exome sequencing

Whole-exome capture was performed using the Agilent SureSelect Human All Exon V6 Kit (Agilent, Santa Clara, CA, USA) according to the instructions provided by the manufacturer. Libraries were sequenced on the Illumina NovaSeq platform (Illumina Inc., San Diego, CA, USA), and an average size of 180–280 bp paired-end reads was generated. WES was performed at an average depth of 220x for tumors and normal tissues. Following quality control using FASTQC, trimmed sequencing reads were aligned to the human reference genome GRCh37 (UCSC hg19) using BWA (v0.1.22).^100^ Duplicate reads were removed using Picard (v1.4.5) (http://broadinstitute.github.io/picard/).

MuTect (v1.1.4) (http://www.broadinstitute.org/cancer/cga/mutect) and Strelka (v1.0.13)^101^ were used to predict somatic single-nucleotide variants (SNVs) and small insertions and deletions (INDELs) in SCLC tumors, with the corresponding adjacent tissues (or PBMCs or oral epithelium) used as controls. Filtering was performed considering the sequencing depth and mutated read counts using the GATK-Variant Filtration. Filtered somatic mutations were functionally annotated using the ANNOVAR software. MutSigCV (v1.41)^102^ was employed to evaluate the significance of the mutated genes, and the results were manually constrained using the chi-square test for variants and coding sequence length. We also considered the ratio of synonymous/nonsynonymous mutations (Ka/Ks) as an indicator of selection pressure.

Somatic single-base substitutions (SBSs) along with adjacent base pairs were used to generate 96 trinucleotide-contextualized mutational signatures. We used the R package MutationalPatterns (v1.8.0)^74^ to decipher mutational signatures, which were compared with known cancer-associated signatures from the COSMIC database and carcinogen signatures reported by Kucab et al.^75^ We applied the nonnegative matrix factorization algorithm in MutationalPatterns to obtain de novo signatures and the optimal factorization rank, independent of recognized signatures. Cosine similarity analysis and reconstruction were used to compare the published signatures and de novo signatures using the R package Palimpsest (v2.0.0).^103^ We also explored doublet base substitution (DBS) and small insertion and deletion signatures in 111 samples using MutationalPatterns.

### Whole-transcriptome sequencing

Total RNA was extracted from freshly frozen tumors and normal lung tissue from patients with treatment-naïve SCLC (Supplementary Table 1). A total of 3 μg of RNA per sample was applied to generate sequencing libraries using the NEBNext® UltraTM RNA Library Prep Kit for Illumina® (NEB, USA), which were sequenced on an Illumina HiSeq platform, generating 150 bp paired-end reads. After quality control, the sequenced reads were aligned to GRCh37 (hg19) UCSC-annotated transcripts using HISAT2 (v2.0.5).^104^ The transcripts were then assembled and counted using featureCounts (v1.5.0),^105^ and gene annotations were obtained from GeneCode. Differentially expressed genes between the tumor/normal and smoker/nonsmoker groups were analyzed using the R package DESeq2 (v1.16.1).^106^ We used P.adjust < 0.01 and |log2FC | > 2 as cutoffs for tumor/normal samples (*P* value < 0.05 for smokers/nonsmokers) to identify significantly differentially expressed genes. Enrichment analysis was performed using the clusterProfiler^107^ R package. The ESTIMATE^108^ R package was used to assign a score to each sample, dividing tumors into “hot” and “cold” groups. We used the normalized enrichment score from the GSVA^109^ R package to perform single-sample GSEA. GSVA also helps in identifying NE samples by calculating NE and non-NE scores in parallel, the genes of which were provided by Wei et al.^48^. The CIBERSORT^110^ and xCell^46^ R packages were used to estimate the percentages of different immune cells in each sample.

We used the STAR-Fusion package (v1.2.0)^52^ to detect and filter the fusion genes. Further filtration was performed using the STAR-Fusion solution (FusionInspector), excluding fusion genes found in paired normal tissues and VDJ regions. We also considered the number of total reads that supported fusion. Two of the identified fusion genes were further validated using RT‒PCR.

### Analysis of ASEs

ASEs were analyzed using rMATS (v4.0.1).^54^ The RNA-seq reads were mapped to the human genome assembly GRCh38 using the STAR aligner.^111^ The junction count outputs were used for further analysis. Splice variants were compared between tumors and normal tissues. The inclusion and exclusion junction reads from replicates were averaged and used to calculate the PSI score for each splice site. Following the calculation, ASEs that met the following criteria were considered significant: false discovery rate (FDR) < 0.01, total related reads in either sample average (tumors vs normal tissues) ≥ 5 in either splicing form, inclusion level difference ≥0.05 or ≤-0.05, and consistent inclusion difference in each sample (tumors vs normal tissues). The frequently detected ASEs were verified via RT‒PCR via cDNA templates and primers (Supplementary Table 16). *FAK* transcripts were amplified via PCR using primers listed in Supplementary Table 16 and subsequently cloned and inserted into Flag-tagged pcDNA3.1 vectors using the Vazyme C115 ClonExpress® Ultra One Step Cloning Kit (Vazyme Biotech, Nanjing, China) through homologous recombination. Plasmid construction was confirmed by Sanger sequencing prior to functional assays.

### 16S rRNA gene sequencing

The amount of bacterial DNA was quantified using the Femto Bacterial DNA Quantification Kit (Zymo Research, Irvine, CA, USA), with the *Escherichia coli* genome used as a standard. Human DNA was quantified using real-time PCR with the primers β-Actin_Forward and β-Actin_Reverse, along with a probe (CTGTGCTATCCCTGTACGCCTCTGGC-VIC) and TaqMan™ Fast Advanced Master Mix (Thermo Fisher Scientific, Waltham, MA).

Total genomic DNA was extracted from tissues using an AllPrep DNA/RNA Mini Kit (Qiagen, Valencia, CA, USA). Variable region 4 (V4) of the 16S rRNA gene was amplified using primers 515FB and 806RB with Phusion High-Fidelity PCR Master Mix (New England Biolabs, Ipswich, MA, USA). The PCR products were separated on a 2% agarose gel and purified using a GeneJET Gel Extraction Kit (Thermo Fisher Scientific). Libraries were prepared using the Ion Plus Fragment Library Kit (Thermo Fisher Scientific) and sequenced on the Ion S5 XL platform, generating 400 bp single-end reads.

### Microbiota data analysis

The sequencing reads were demultiplexed, and the barcodes and primer sequences were removed via Cutadapt (v1.9.1).^112^ Chimeras were filtered out via the UCHIME algorithm^113^ in comparison with the Silva database.^114^ The taxonomy of each read was assigned via the Ribosomal Database Project Classifier (v2.13)^115^ with a cutoff of 0.8.

Total sum scaling was applied to normalize the microbiome data. Alpha and beta diversity were analyzed after rarefaction to 20,000 reads, and the Shannon index, observed genera, and Bray‒Curtis distances were determined via the phyloseq R package (v1.30.0).^116^ Differential clustering of the microbial communities was assessed via PERMANOVA with the Adonis function in the vegan R package (v2.5-7).^117^ The paired Wilcoxon signed-rank test was used to compare the tumor and normal groups. Multiple comparisons were corrected via the Benjamini‒Hochberg FDR algorithm,^118^ with a significance level of 0.05.

### In situ hybridization and immunohistochemistry assays

To visualize the splicing variants of *FAK* on slides, BaseScope Duplex assays were performed using FFPE samples and BaseScope probes according to the protocols provided by the supplier (Advanced Cell Diagnostics, Newark, CA, USA). IHC was performed using an anti-phospho-FAK (Y397) antibody as previously described,^55^ and the immunoreactivity score (IRS) was calculated as IRS (0–12) = RP (0–4) × SI (0–3), where RP is the percentage of positive stained cells and SI is the staining intensity.

### Structure prediction and analysis

The structure of FAK was downloaded from the AlphaFold protein structure database^119^ (https://alphafold.ebi.ac.uk/). The three mutant structures (FAK^6^, FAK^7^, and FAK^6,7^) were predicted via AlphaFold. Sequence alignments were performed via MultAlin^120^ and visualized via ENDscript.^121^ All structural figures were generated via PyMOL.

## Spatial single-cell proteomics analysis

### Antibody conjugation

Carrier-free antibodies were conjugated to the barcodes via commercial reagents purchased from Akoya Bioscience (Marlborough, MA, USA). Amicon Ultra 50 K centrifugation filters (Millipore, Darmstadt, Germany) were washed with 500 μL of filter blocking solution (Akoya Biosciences, Marlborough, USA) and centrifuged at 12,000 × *g* for 2 min. A total of 50 μg of antibody was added to the filter and centrifuged at 12,000 × *g* for 8 min. Next, 260 μL of antibody reduction master mix (Akoya Biosciences) was added to the filter and incubated at room temperature for 30 min. After centrifugation at 12,000 × *g* for 8 min, the filter was washed three times with 450 μL of conjugation buffer (Akoya Biosciences). The barcode was resuspended in 10 μL of nuclease-free water, complemented with 210 μL of conjugation buffer, and added to the filter. After incubation at room temperature for 2 h, the filter was centrifuged at 12,000 × *g* for 8 min and washed three times with 450 μL of purification solution (Akoya Biosciences). The purified antibody was resuspended in 100 μL of antibody storage solution (Akoya Biosciences) and stored at 4 °C.

### PhenoCycler-Fusion multiplex tissue staining and imaging

FFPE tissue sections mounted on poly-L-lysine-coated coverslips were stored at 4 °C. The tissue sections were incubated at 65 °C for at least 2 h, deparaffinized, and rehydrated. Heat-induced epitope repair was performed via alkaline antigen retrieval (pH = 9) at 110 °C for 20 min. The sections were cooled to room temperature, soaked in hydration buffer (Akoya Biosciences) for 2 min, and equilibrated with staining buffer (Akoya Biosciences) for 30 min. The antibodies were combined at the dilutions indicated in Supplementary Table 16, added to the sections, and incubated at room temperature for 3 h. After staining, the sections were washed twice with staining buffer, fixed with 1.6% formaldehyde solution (Thermo Fisher Scientific) for 10 min and washed three times with PBS. The sections were incubated with ice-cold methanol at 4 °C for 5 min and washed three times with PBS. The sections were fixed with fresh fixation reagent (Akoya Biosciences) for 20 min and then subjected to cyclic imaging immediately.

### PhenoCycler-Fusion image and data analysis

Whole-tissue cell segmentation was performed via the StarDist plugin in QuPath.^122^ Spatially defined tissue regions were manually annotated as previously described.^123^ In accordance with the tissue morphology and distribution of the tumor cell markers CD56 and EPCAM, as well as the immune cell marker CD45, the tumor core (referred to as “tumor”), tumor invasion margin (Rim) and adjacent normal (N) tissues were manually annotated via QuPath software. StarDist, a deep learning-based method for nucleus detection and segmentation, was used with a pretrained model from Akoya Biosciences. Single-cell data, including fluorescence cell marker intensity and spatial coordinates generated on the basis of cell segmentation, were used for downstream analysis. Arcsinh transformation was applied to the raw fluorescence intensity values to normalize the PhenoCycler-Fusion single-cell data. A self-organizing map method was used for the cluster analysis, and the cells were annotated according to the average expression of the different markers in each cluster.

### Organoid culture

Human SCLC tumors were sectioned into 5 mm^3^ pieces and incubated with digestion buffer containing 1.0 mg/mL collagenase I (Gibco, California, USA) and 0.5 mg/mL collagenase IV (Gibco, California, USA) in DMEM/F12 (Gibco) for 1 h at 37 °C, with mechanical pipetting every 15 min. The digested samples were filtered through 70 μm cell strainers. After red blood cells were lysed in ammonium chloride-potassium lysis buffer, the remaining cells were collected by centrifugation and resuspended in ice-cold Matrigel (Corning, USA). The mixture was plated into a 48-well tissue culture plate (40 μL drop containing 50,000 cells) and incubated for 15 min. Prewarmed organoid culture medium prepared according to a previously reported method^66^ was added to the culture.

Human SCLC organoids were seeded in 96-well plates and cultured for 24 h. The cells were treated with 0, 0.6, 3, 15, or 75 μM PF562271 for 72 h. Organoid viability was quantified via a Cell Counting Kit-8 (CCK-8) proliferation assay. Representative organoid images were taken at 72 h posttreatment.

### Screening of cytokines via the Multiplex Luminex System

A Magnetic Luminex® Assay (R&D Systems, Minneapolis, Canada) was used following the manufacturer’s instructions for the detection of multiple cytokines in the plasma. Briefly, microparticles, plasma, and standards were incubated in a 96-well plate precoated with cytokine-specific antibodies. After 2 h of incubation, the plate was washed and incubated with a biotinylated antibody cocktail specific to the cytokines of interest for 1 h. A second wash was performed to remove the unbound biotinylated antibodies. Furthermore, a streptavidin‒phycoerythrin conjugate was added to each well to bind to the biotinylated antibodies. After a final wash, the microparticles were resuspended in buffer and assessed via a Luminex® 200™ Analyzer (R&D Systems, Minneapolis, MN, Canada).

### Cell proliferation analysis

DMS114 and H446 cells were seeded at 2.5 × 10^5^ cells/well into 6-well plates for the IncuCyte live cell assay. The cells were treated with the FAK inhibitor PF562271 at 0, 2.5, 5, or 10 μM and were imaged every 6 h for 72 h. Cell proliferation was determined as percent confluence from phase images and was analyzed by IncuCyte image analysis software.

H82 and H524 cells were seeded at 1 × 10^4^ cells/well into 96-well plates for the CCK-8 assay. The cells were treated with the FAK inhibitor PF562271 at 0, 1, 2.5, or 5 μM for 0, 24, 48, 72 or 96 h, and CCK-8 solution (Vazyme, Nanjing, China) was added to each well. After 2 h of incubation, the absorbance at 450 nm was measured with a microplate reader. The cell viability was calculated as [(As-Ab)/(Ac-Ab)] × 100%, where As is the absorbance of the experimental samples, Ab is the absorbance of the blank plates and Ac is the absorbance of the control plates.

### Western blot and tyrosine kinase activity assays

Proteins were extracted from frozen tissues or cells and subjected to Western blotting with the indicated antibodies (Supplementary Table 16). For kinase activity, proteins were harvested from EGFP-*FAK*-expressing HEK293 cells, purified via protein A/G agarose (Santa Cruz Biotechnology) and anti-GFP antibody-mediated immunoprecipitation and analyzed with a Universal Tyrosine Kinase Assay Kit (Clontech, Palo Alto, CA).^55^

### Animal study

To establish patient-derived xenograft (PDX) models, patient-derived tumors were obtained with written informed consent, and approximately 30 mg of tissue fragments were implanted subcutaneously into the flank region of nonobese diabetic (NOD)/ShiLtJGpt-Prkdc^em26Cd52^Il2rg^em26Cd22^/Gpt (null; NCG) male mice (4–6 weeks old) via a trocar. Successfully established models were passaged and banked after three passages in mice. H82 cells (3 × 10^6^) were implanted subcutaneously into the flank region of NCG mice. The tumor size was measured every other day with an electronic caliper, and when the tumors reached 50 mm^3^, the mice were randomized into two or three groups and treated with the vehicle control or PF562271. The vehicle control (0.5% CMC-Na) was prepared by dissolving 5 g of carboxymethyl cellulose sodium (CMC-Na) in 1 L of sterile water, stirring for 2–4 h at room temperature, and then autoclaving (121 °C, 15 min) for sterilization. PF562271 suspensions were formulated prior to administration by suspending the compound in the autoclaved 0.5% CMC-Na vehicle at concentrations of 5 mg/mL or 15 mg/mL. Each suspension was briefly vortexed and sonicated to ensure homogeneity. The mice received oral gavage at 10 μL/g body weight, with the control group receiving 0.5% CMC-Na vehicle alone and the treatment group receiving PF562271 at 50 or 150 mg/kg/day, 5 days per week for 2–6 weeks. The animals were sacrificed when the tumors reached 2 cm or if the mice appeared moribund, and the tumor tissues were excised, photographed, and analyzed.

### Quantification and statistical analysis

To compute the MHC-I scores of the cancer cell clusters, individual cells were scored via the AddModuleScore function implemented in the Seurat package, which calculates the average expression levels of selected genes (HLA-A, HLA-B, and HLA-C) at the single-cell level. On the basis of the MHC-I scores, we defined the SCLC clusters into three subtypes: MHC-I-low (MHC-I scores < −0.1), MHC-I-moderate (−0.1 ≤ MHC-I scores ≤ 0.1), and MHC-I-high (MHC-I scores > 0.1). All the statistical analyses were conducted via GraphPad Prism 5 (GraphPad Software, La Jolla, CA, USA) and R software (v4.3.2). Statistically significant differences were determined by Fisher’s exact test, Student’s *t* test, the Mann‒Whitney test, or the Wilcoxon rank sum test, as indicated. Survival curves for each group were generated via the Kaplan‒Meier method and log-rank test. *P* values less than 0.05 were considered statistically significant.

### Material availability

The requests for resources and reagents should be directed to and will be fulfilled by the lead contact, Guang-Biao Zhou (gbzhou@cicams.ac.cn).

## Supplementary information

SUPPLEMENTAL MATERIAL

Table S1

Table S2

Table S3

Table S4

Table S5

Table S6

Table S7

Table S8

Table S13

Table S14

Table S15

## Data Availability

All the raw data involved in this study have been deposited at the National Genomics Data Center of China (https://ngdc.cncb.ac.cn/) under accession numbers PRJCA023692 (human SCLC scRNA-seq data: HRA007131, HRA007382), PRJCA024141 (human SCLC WES data: HRA007141), PRJCA024130 (human SCLC bulk RNA-seq data: HRA007125), and PRJCA025292 (human SCLC 16S rRNA gene-seq data: CRA016047), which are publicly available as of the date of publication. The accession numbers are listed in Supplementary Table 16. This paper analyzes existing, publicly available data^124^ from the European Nucleotide Archive (ENA). These accession numbers for the datasets are listed in Supplementary Table 16. Any additional information required to reanalyze the data reported in this paper is available from the lead contact upon reasonable request.

## Code availability

All the raw data involved in this study have been deposited at the National Genomics Data Center of China (https://ngdc.cncb.ac.cn/) under accession numbers PRJCA023692 (human SCLC scRNA-seq data: HRA007131, HRA007382), PRJCA024141 (human SCLC WES data: HRA007141), PRJCA024130 (human SCLC bulk RNA-seq data: HRA007125), and PRJCA025292 (human SCLC 16S rRNA gene-seq data: CRA016047), which are publicly available as of the date of publication. The accession numbers are listed in Supplementary Table 16. This paper analyzes existing, publicly available data^124^ from the European Nucleotide Archive (ENA). These accession numbers for the datasets are listed in Supplementary Table 16. Any additional information required to reanalyze the data reported in this paper is available from the lead contact upon reasonable request.
