# Supplementary material for: Characterization of the extrinsic and intrinsic signatures and therapeutic vulnerability of small cell lung cancers
Source: Signal Transduct Target Ther. 2025 Sep 10;10:290. doi: 10.1038/s41392-025-02378-6 (PMC12420805; doi:10.1038/s41392-025-02378-6)
Supplement: Supplementary file 1 — SUPPLEMENTAL MATERIAL [file 41392_2025_2378_MOESM1_ESM.docx]

Supplementary Materials for

Characterizing extrinsic and intrinsic signatures and therapeutic vulnerability of patients with small cell lung cancer

Gui-Zhen Wang^1^*, Zheng Wang^1^*, Shi-Hao Bai^2^*, Yun Tan^3^*, Wen-Zhao Zhong^4^*, Guo-Gui Sun^5^*, Yu-Tao Liu^1^*, Bo Pan^6^, Chen Huang^4^, Di Wang^1^, Bei-Bei Sun^1^, Dong-Ni Chen^7^, Bin Zhang^8^, Yong-Chun Zhou^9^, Sheng Li^10^, Xiang-Wei Zhang^11^, Si-Chong Han^1^, Fu-Ying Yang^1^, Xue-Yan Shi^1^, Xiao-Liang Jie^1^, Yu-Ke Shen^1^, Li-Jun Liang^1^, Zhe-Sheng Wen^7^, Li Zhang^12^, Ming-Kun Li^12^, Na Wang^13^, Jin-song Liu^13^, Ying Dong^14^, Man-Li Wang^15^, Yan Wang^1^, Chang-Li Wang^8^, Da-Wei Xie^1^, Ze-Guang Han^2^, Jian-Ming Ying^1^, Chong Chen^15^, Yun-Chao Huang^9^, Hong-Bin Ji^16^, Yuan-Yuan Zhang^1^, Yan Yu^6#^, Guang-Biao Zhou^1,17#^

#Corresponding authors. Email: [gbzhou@cicams.ac.cn](mailto:gbzhou@cicams.ac.cn) (GBZ) or [gpyuyan@163.com](mailto:gpyuyan@163.com) (YY).

**This PDF file includes:**

Figures. S1 to S20

Tables S9 to S12, and S16

Uncropped images of Western Blots

**Other Supplementary Materials for this manuscript include the following:**

Tables S1 to S8, and S13 to S15

Figure. S1.

**Figure S1. Study design of this work.** Total number of patients whose samples were analyzed in this study is shown at the middle line of the figure.

Figure. S2.

**Figure S2. scRNA-seq analysis of SCLCs, Related to Figure 1.** (**a**) UMAP plot of all cells clustered and color coded by patients and tissues. (**b**) Dot plot of selected marker genes in B and T/NK cell lineage. Dot size and color indicate the fraction of expressing cells and normalized expression levels, respectively. (**d**) Boxplots representing the relative proportions of each cell type in the patient samples. Comparisons were made among different sample types. Sample type: P, peripheral blood mononuclear cell; N, adjacent normal lung tissue; T, tumor tissue; L, lymphoid tissue; Treatment: N, no; Y, yes. (**d,** **e**) Comparisons of indicated cell types among different stages classified by the TNM staging methods (**d**) and outcomes of neoadjuvant therapy (**e**). Boxplots representing the proportions of each cell type in patient samples. *P* values, Wilcoxon rank-sum test. *, *P* < 0.05; **, *P* < 0.01.

Figure. S3.

**Figure S3. Immune cells in SCLC, detected using PhenoCycler-Fusion 2.0.** (**a**) Fifteen cell types were identified based on the expression of 25 proteins, with a heatmap illustrating the levels of these 25 proteins across different cell types. (**b-d**) Spatial distribution of immune and cancer cells in tumor samples with patient characteristics listed at the bottom of each image. Selected proteins are shown in (**b**), and proportions of indicated cell types in patients with indicated clinical characteristics are shown in (**c**) and (**d**). (**e, f**) Spatial distribution of immune and cancer cells in tumor samples with patient characteristics listed at the bottom of each image. Selected proteins are shown in (**e**), and proportions of indicated cell types in patients with indicated clinical characteristics are shown in (**f**). *P* values in **c, d**, **f**, Student’s t test. *, *P* < 0.05; **, *P* < 0.01.

Figure. S4.

**Figure S4. Macrophage/monocyte and NK cells in SCLC, Related to Figure 1 and 2.** (**a**) Gene set enrichment analysis (GSEA) plots for indicated gene sets in tumor cells of smokers and non-smokers. (**b**) Comparison of differentially expressed genes in macrophages from tumor samples of smokers and non-smokers. (**c**) Boxplots representing the relative proportions of each macrophage/monocyte population. Comparisons were made among different sample types. Sample type: P, peripheral blood mononuclear cell; N, adjacent normal lung tissue; T, tumor tissue; L, lymphoid tissue; Treatment: N, no; Y, yes. (**d**) GSEA of genes differentially expressed between Mφ-S100A12 and Mφ-SPP1. Gene sets related to antigen processing and presentation and response to chemokine are shown. (**e**) IHC analysis of NK cells in FFPE samples of patients with untreated SCLC using an anti-CD94 antibody.

Figure. S5.

**Figure S5.** ***HNF4A*, copy number variations, and antigen presentation pathway in cancer cell, Related to Figures 1, 3, and 4.** (**a**) *HNF4A* in cancer cells of our scRNA-seq data. (**b**) *HNF4A* in tumor tissues of our bulk RNA-seq data. *P* > 0.05 (not indicated), Student’s *t* test. (**c**) Heatmap shows large-scale copy number variations (CNVs) in malignant cells from 39 SCLC patients. The inferred CNVs were deduced for each single cell based on the average expression profiles across chromosomal intervals. Normal epithelia from adjacent normal lung tissues were used as references. (**d**) The expression of MHC molecules in cell lines of SCLC and other cancers. Data were obtained from the Cancer Cell Line Encyclopedia database. *P* values were determined using the Wilcoxon rank-sum test. ** *P* < 0.01, *** *P* < 0.001. (**e**) Comparison of MHC-I/II gene enrichment score in indicated cells at different stages, with samples harvesting information and number of patients shown under the images. (**f**) Comparison of MHC-I/II gene enrichment score in indicated cells among patients. The response and number of patients are shown under the images. (**g**) Analysis of cell-cell interaction signals involving MHC-I and MHC-II among cancer cells and immune cells in each patient using the CellChat algorithm, with interaction scores between different cell types are presented in heatmap plots.

Figure. S6.

**Figure S6. Pathway enrichment in SCLCs, Related to Figure 5.** (**a**) GO and KEGG enrichment of upregulated genes (left) / down-regulated genes (right) in tumor samples. Top 10 significant pathways are shown. Dot size and color indicate the expression level and gene number of the pathways, respectively. (**b**) Top 5 upregulated (upper) / down-regulated (lower) Gene Ontology Biological Process (GOBP) pathways between tumors and adjacent tumors by GSEA. (**c)** Gene Ontology (GO) and Kyoto Encyclopedia of Genes and Genomes (KEGG) enrichment of upregulated genes (left) / down-regulated genes (right) in tumor samples of smoker patients in comparison with nonsmoker patients’ tumors.

Figure. S7.

**Figure S7.** **Dysregulated genes from bulk RNA sequencing data, Related to Figure 5.** (**a**) Comparison of differentially expressed genes/pathways between tumor and normal control tissues. (**b**) Volcano plot of differentially expressed genes relevant to MHC pathway and neuroendocrine genes (cutoff: p.adjust < 0.05 & |log2FC| > 0.5). (**c**) Significant differential immune infiltrations between tumor and adjacent tissue samples. *P* value, Nonparametric test. *, *P* < 0.05; **, *P* < 0.01; ***, *P* < 0.001. (**d**) Heatmap of differentially expressed genes related to cytokines and chemokines. (**e**) Serum concentration of indicated chemokines were measured by the multiplex Luminex System. *P* value, Student’s *t* test. (**f**) Comparison of tumor mutation burden (TMB) between hot tumors and cold tumors. *P* value, Student’s *t* test.

Figure. S8.

**Figure S8. Potential fusion genes found in bulk RNA-seq, Related to Figure 5.** (**a**) Fusion genes found in individual patients. The ones marked with * have been reported in the previous study^1^. (**b)** Distribution of gene fusions across genome. Circos plot representing the fusions between different chromosomes. (**c**) Fusion gene *ARID1B-ZDHHC14* in the tumor-normal paired samples of SCLC patient was detected by RT-PCR assays. (**d**) Fusion gene *CSNK1D-CCDC57* in the tumor-normal paired samples of SCLC patient was detected by RT-PCR assays. T, tumor tissue; N, adjacent normal lung tissue.

Figure. S9.

**Figure S9. Alternative splicing events (ASEs) of SCLCs, Related to Figure 5.** **(a)** Study design for identification of high frequency ASEs in SCLCs. **(b)** The five classes of ASEs found in the patients. **(c)** Number of genes bearing ASEs. (**d**) The splicing variants of REST (svREST) at mRNA and protein levels. CDS, coding sequence. (**e**) The percent spliced-in (PSI) values of *REST* transcripts containing the 50 bp box in tumor- and counterpart normal lung-tissues. *P* value, Student’s *t* test. (**f**) *vREST* in the tumor-normal paired samples of 5 patients was detected by RT-PCR assays. T, tumor tissue; N, adjacent normal lung tissue. (**g**) s*vREST* in 4 SCLC cell lines detected by RT-PCR assays. (**h - i**) The frequency of *FAK^6/7^* in combined SCLC and SCLC of this cohort (**h**) and this cohort together with Liu et al’s cohort^2^ (**i**). *P* value, Student’s *t* test.

Figure. S10.

**Figure S10. FAK^6/7^ in SCLCs, Related to Figure 5.** (**a**) Structure-based sequence alignment of FAK and its variants. Only the regions containing FERM and Kinase domain (35-686) are shown. The secondary structures are noted upon the sequences. The two variant sites are shown in yellow box. **(b)** Representative Sanger sequencing results of the RT-PCR products detecting *FAK* variants using tissue samples of indicated patients. **(c)** Representative Sanger sequencing results of the RT-PCR products cloned into the pCE2-TA/Blunt-Zero plasmid.

Figure. S11.

**Figure S11. FAK splicing variants in SCLC cell lines, Related to Figure 6.** **(a)** Percent spliced-in values of Box 6 and Box 7 in indicated cell lines. Data were downloaded from the Cancer Cell Line Encyclopedia (CCLE) data on the Cancer Dependency Portal (DepMap; https://depmap.org/portal/ccle/)^3^. **(b)** *FAK* variants in indicated cell lines detected by RT-PCR. **(c)** The expression of phosphorylated FAK (p-FAK) and FAK in cell lines detected by Western blot. Results are representative of three independent experiments. **(d)** DMS114 cells were transfected with plasmids containing *FAK* variants, lysed, and subjected to Western blot assays using indicated antibodies. Results are representative of three independent experiments. **(e)** Relative tyrosine kinase activity of FAK variants purified from HEK293 cells that were transfected with corresponding plasmids. Data of FAK variants were normalized and are presented as fold change relative to Flag control samples; mean ± s.d. from three biologically independent replicates per condition. *P* value, two-tailed unpaired *t*-test. **(f)** The expression level of p-FAK in DMS114 cells transfected with indicated plasmids at indicated volume (μg). **(g)** Effects of AKT and mTOR inhibitors on cell proliferation of cells with *FAK* or its splicing variants.

Figure. S12.

**Figure S12. Effects of FAK inhibitors on SCLC cells, Related to Figure 7.** (**a**) Inhibition rate of si*FAK* on cell proliferation in lines with *FAK* (DMS114) or *FAK^6,7^* (H446). (**b**) Inhibition rate of PF562271 on cell viability of lines with *FAK* or splicing variants. **(c)** Effects of PF562271 on cell proliferation of ling with *FAK* or *FAK^6,7^*. **(d)** Effects of PF562271 on cell proliferation of lines with *FAK^6,7^*. **(e, f)** Effects of PF562271 (**e**) and PF563228 (**f**) on cell cycle distribution in cells with *FAK* or *FAK^6,7^*. Data are presented as mean ± s.d. from three biologically independent replicates per condition. *P* value determined by two-tailed unpaired *t*-test. **(g, h)** Effects of PF562271 and PF573228 (1 μM) on colony forming activity of DMS114 and H446 cells. *P* values, Student’s *t* test. **(i, j)** Effects of PF562271 and PF573228 (1 μM) on migration activity of DMS114 and H446 cells, detected by transwell assays. *P* values, Student’s *t* test. (**k, l**) Effects of FAK inhibitors on p-FAK expression in SCLC cells. The cells were treated with PF562271 (k) or PF573228 (l) for 48 h, lysed, and subjected to Western blot using indicated antibodies.

Figure. S13.

**Figure S13. Microbiome in tumor and normal tissues of SCLCs, Related to Figure 8.** (**a**) Bacterial DNA proportion in tumor and normal tissues. *P* value, Student’s *t* test. Shannon index (**b**) and Observed genera (**c**) of the microbiome. In A-C, paired Wilcoxon signed-rank tests were used. (**d**) PCoA based on Bray-Curtis distance between microbiomes profiled at the genus level. R^2^ and *P* values from PERMANOVA tests were shown. In (**a - d**), tumor and normal tissues from the same individual were indicated by connecting lines. (**e**) Relative abundances of the top ten phyla. (F) Relative abundances of the top 30 genera.

Figure. S14.

**Figure S14.** **Pathway enrichment in recurrently mutated genes in SCLC, Related to Figure 8.** (**a, b**) Gene Ontology (GO) (**a**) and Kyoto Encyclopedia of Genes and Genomes (KEGG) (**b**) analyses of mutated genes identified in this study.

Figure. S15.

**Figure S15. Mutations in calcium, sodium and other ion channel genes in SCLCs, Related to Figure 8.** This figure was created using BioRender (www.biorender.com).

Figure. S16.

**Figure S16. Frequently somatically mutated genes identified in this cohort of SCLCs, Related to Figure 8.** Somatic exonic mutations are shown in changes in amino acids. Mutation rate of each gene was indicated.

Figure. S17.

**Figure S17.** **Expression of *Cyclin D* in *TP53* and *Rb1* wild-type tumors, Related to Figure 8.** (**a**) Expression of *Cyclin D1*, *D2* and *D3* between *Rb1* wild-type (wt) tumors and *Rb1* mutant tumors in SCLC of our cohort, and cohorts of George et al^4^ and Jiang et at^5^. (**b**) Expression of *Cyclin D1*, *D2* and *D3* between *TP53* wild-type (wt) tumors and *TP53* mutant tumors in our cohort, George et al^4^ and Jiang et at^5^. *P* value, Student’s *t* test.

Figure. S18.

**Figure S18. Comparison of** **mutations in Chinese SCLCs and Caucasian SCLCs, Related to Figure 8.** Fisher's exact test. *, *P* < 0.05; **, *P* < 0.01; ***, *P* < 0.001.

Figure. S19.

**Figure S19. Mutational signatures of each patient, Related to Figure 8.** Single base substitutions

(SBS) of the patients are shown.

Figure. S20.

**Figure S20.** **Signatures of environmental carcinogens in SCLC, Related to Figure 8.** (**a**) Cosine similarity heatmap of the 4 de novo signatures and published single base substitution (SBS) signatures. (**b**) Carcinogen signatures in smoker and nonsmoker patients. Tumors are arranged from left to right in the top track.

Table S9.

| **Characteristics** | **Patients, n (%)** |
| --- | --- |
| ***Total*** | 154 (100) |
| Gender |  |
| Male | 109（70.8） |
| Female | 45（29.2） |
| Age |  |
| <60 | 89（57.8） |
| ≥60 | 65（42.2） |
| Smoking |  |
| Smoker | 107（69.5） |
| Non-smoker | 44（28.6） |
| n.d. | 3（1.9） |
| Histology |  |
| SCLC | 143（92.9） |
| Combined SCLC | 11（7.1） |
| TNM stage |  |
| IA-IIB | 82（53.2） |
| IIIA-IV | 53（34.5） |
| n.d. | 19（12.3） |
| ***RNA-seq*** | 45 |
| Gender |  |
| Male | 40（88.9） |
| Female | 5（11.1） |
| Age |  |
| <60 | 21（46.7） |
| ≥60 | 24（53.3） |
| Smoking |  |
| Smoker | 36（80.0） |
| Non-smoker | 7（15.6） |
| n.d. | 2（4.4） |
| Histology |  |
| SCLC | 37（82.2） |
| Combined SCLC | 8（17.8） |
| TNM stage |  |
| IA-IIB | 13（28.9） |
| IIIA-IV | 13（28.9） |
| n.d. | 19（42.2） |
| ***RT-PCR-seq*** | 37 |
| Gender |  |
| Male | 29 (78.4) |
| Female | 8 (21.6) |
| Age |  |
| <60 | 21 (56.8) |
| ≥60 | 16 (43.2) |
| Smoking |  |
| Smoker | 28 (75.7) |
| Non-smoker | 7 (18.9) |
| n.d. | 2 (5.4) |
| Histology |  |
| SCLC | 26 (70.3) |
| Combined SCLC | 11 (29.7) |
| TNM stage |  |
| IA-IIB | 17 (45.9) |
| IIIA-IV | 19 (51.4) |
| n.d. | 1 (2.7) |
| ***Basescope/FISH*** | ***56*** |
| Gender |  |
| Male | 36 (64.3) |
| Female | 20 (35.7) |
| Age |  |
| <60 | 36 (64.3) |
| ≥60 | 20 (35.7) |
| Smoking |  |
| Smoker | 37 (66.1) |
| Non-smoker | 19 (33.9) |
| Histology |  |
| SCLC | 56 (100) |
| Combined SCLC | 0 (0) |
| TNM stage |  |
| IA-IIB | 37 (66.1) |
| IIIA-IV | 19 (33.9) |
| ***RT-PCR and IHC*** | 99 |
| Gender |  |
| Male | 63 (63.6) |
| Female | 36 (36.4) |
| Age |  |
| <60 | 61 (61.6) |
| ≥60 | 38 (38.4) |
| Smoking |  |
| Smoker | 63 (63.6) |
| Non-smoker | 35 (35.4) |
| n.d. | 1 (1) |
| Histology |  |
| SCLC | 99 (100) |
| Combined SCLC | 0 (0) |
| TNM stage |  |
| IA-IIB | 65 (65.7) |
| IIIA-IV | 34 (34.3) |

**Table S9. Baseline demographic characteristics of patients whose samples were tested for *FAK* splicing variants.**

Table S10.

| **Characteristics** | **Total,**  **n (%)** | ***FAK*,**  **n (%)** | **Splicing variants (n, %)** | | | ***P*** |
| --- | --- | --- | --- | --- | --- | --- |
|  |  |  | ***FAK^6,7^*** | ***FAK^6^*/*FAK^7^*** | **Combined** |  |
| Total | 154 (100) | 35 (22.7) | 89 (57.8) | 30 (19.5) | 119 (77.3) |  |
| Sex |  |  |  |  |  |  |
| Male | 109（70.8） | 27 (24.8) | 57 (52.3) | 25 (22.9) | 82 (75.2) | 0.346 |
| Female | 45（29.2） | 8(17.8) | 32 (71.1) | 5 (11.1) | 37 (82.2) |  |
| n.d. |  |  |  |  |  |  |
| Age, y |  |  |  |  |  |  |
| <60 | 89（57.8） | 23 (25.8) | 53 (59.6) | 13 (14.6) | 66 (74.2) | 0.280 |
| ≥60 | 65（42.2） | 12 (18.5) | 36 (55.4) | 17 (26.1) | 53 (81.5) |  |
| n.d. |  |  |  |  |  |  |
| Smoking |  |  |  |  |  |  |
| Smoker | 107（69.5） | 24 (22.4) | 60 (56.1) | 23 (21.5) | 83 (77.6) | 0.841 |
| Non-smoker | 46（29.9） | 11 (23.9) | 28 (60.9) | 7 (15.2) | 35 (76.1) |  |
| n.d. | 1（0.6） |  | 1 (100) |  |  |  |
| Histology |  |  |  |  |  |  |
| Small cell carcinoma | 143（92.9） | 33 (23.1) | 82 (57.3) | 28 (19.6) | 110 (76.9) | 0.709 |
| Combined small cell carcinoma with adenocarcinoma | 11（7.1） | 2(18.2) | 7 (63.6) | 2(18.2) | 9 (81.8) |  |
| TNM stage |  |  |  |  |  |  |
| IA-IIB | 82（53.2） | 19 (23.2) | 55 (67.1) | 8 (9.7) | 63 (76.8) | 0.623 |
| IIIA-IV | 52（33.8） | 14 (26.9) | 32 (61.5) | 6 (11.5) | 38 (73.1) |  |
| n.d | 20（13.0） | 2 (10.0) | 2 (10.0) | 16 (80.0) | 18（90.0） |  |

*, [Chi-square test](https://blog.csdn.net/u011955252/article/details/50704459).

**Table S10. *FAK* splicing variants in the 154 treatment-naïve patients with SCLC.**

Table S11.

|  | Patient # | Age | Sex | Smoking | TMN stage | OS (months) | *FAK* | ANYP Type | Experiment  method |
| --- | --- | --- | --- | --- | --- | --- | --- | --- | --- |
| Frozen tissue | 1 | 62 | M | Yes | 1 | 24.83 | *FAK* | Y | RT-PCR-seq |
|  | 2 | 57 | M | Yes | 3 | 64.77 | *FAK^6,7^* | A | RT-PCR-seq |
|  | 3 | 47 | M | No | 4 | 14.37 | *FAK^6,7^* | N | RT-PCR-seq |
|  | 4 | 44 | M | Yes | 3 |  | *FAK^6^/FAK^7^* | Y | RT-PCR-seq |
|  | 5 | 54 | M | Yes | 3 |  | *FAK^6,7^* | A | RT-PCR-seq |
|  | 6 | 53 | F | No | 1 | 0.10 | *FAK^6^/FAK^7^* | N | RT-PCR-seq |
|  | 7 | 48 | M | Yes | 1 | 68.07 | *FAK^6,7^* | N | RT-PCR-seq |
|  | 8 | 70 | M | Yes | 3 |  | *FAK* | P | RT-PCR-seq |
|  | 9 | 60 | F | No | 2 |  | *FAK^6,7^* | N | RT-PCR-seq |
|  | 10 | 62 | M | Yes | 3 |  | *FAK^6,7^* | A | RT-PCR-seq |
|  | 11 | 59 | M | Yes | 2 | 69.23 | *FAK^6,7^* | A | RT-PCR-seq |
|  | 12 | 60 | M | No | 2 | 73.70 | *FAK^6,7^* | A | RT-PCR-seq |
|  | 13 | 77 | F | No | 1 |  | *FAK^6,7^* | A | RT-PCR-seq |
|  | 14 | 67 | M | No | 1 |  | *FAK^6,7^* | A | RT-PCR-seq |
|  | 15 | 73 | F | No | 3 |  | *FAK^6,7^* | A | RT-PCR-seq |
|  | 16 | 65 | M | Yes | 2 | 8.17 | *FAK^6,7^* | N | RT-PCR-seq |
|  | 17 | 47 | M | Yes | 1 |  | *FAK^6,7^* | N | RT-PCR-seq |
|  | 18 | 62 | M | Yes | 3 | 46.20 | *FAK^6,7^* | A | RT-PCR-seq |
|  | 19 | 52 | M | Yes | 3 | 17.27 | *FAK* | P | RT-PCR-seq |
|  | 20 | 57 | M | Yes | 4 |  | *FAK* | Y | RT-PCR-seq |
|  | 21 | 69 | F | Yes |  | 2.93 | *FAK^6,7^* | A | RT-PCR-seq |
|  | 22 | 47 | M | Yes | 1 | 24.70 | *FAK* | A | RT-PCR-seq |
|  | 23 | 50 | M | Yes |  |  | *FAK^6,7^* | A | RT-PCR-seq |
|  | 24 | 45 | M | Yes | 1 |  | *FAK^6,7^* | A | RT-PCR-seq |
|  | 25 | 54 | F | No | 3 | 24.10 | *FAK^6^/FAK^7^* | A | RT-PCR-seq |
|  | 26 | 65 | F | Yes | 1 |  | *FAK^6,7^* | N | RT-PCR-seq |
|  | 27 | 71 | M | Yes | 3 | 12.57 | *FAK^6,7^* | N | RT-PCR-seq |
|  | 28 | 62 | M | Yes | 1 |  | *FAK^6,7^* | A | RT-PCR-seq |
|  | 29 | 57 | M | Yes | 2 |  | *FAK^6,7^* | N | RT-PCR-seq |
|  | 30 | 55 | M | Yes | 3 | 37.33 | *FAK^6^/FAK^7^* | P | RT-PCR-seq |
|  | 31 | 52 | M | Yes | 1 | 72.50 | *FAK* | N | RT-PCR-seq |
|  | 32 | 56 | M | Yes | 4 | 33.10 | *FAK^6,7^* | P | RT-PCR-seq |
|  | 33 | 58 | M | Yes | 3 | 23.63 | *FAK^6,7^* | A | RT-PCR-seq |
|  | 34 | 70 | M | Yes | 3 |  | *FAK^6,7^* | N | RT-PCR-seq |
|  | 35 | 60 | F | No | 3 |  | *FAK^6,7^* | N | RT-PCR-seq |
|  | 36 | 57 | M | Yes | 4 | 4.30 | *FAK^6,7^* | A | RT-PCR-seq |
|  | 37 | 59 | M | Yes | 2 |  | *FAK^6,7^* | N | RT-PCR-seq |
| FFPE | 38 | 56 | M | Yes | 3 | 5.73 | *FAK^6,7^* | A | RT-PCR/ IHC/ Basescope |
|  | 39 | 59 | M | No | 1 | 75.30 | *FAK* | P | RT-PCR/ IHC |
|  | 40 | 71 | M | Yes | 1 | 61.47 | *FAK^6,7^* | A | RT-PCR/ IHC/Basescope |
|  | 41 | 52 | M | Yes | 3 |  | *FAK^6,7^* | N | RT-PCR/ IHC/Basescope |
|  | 42 | 65 | F | No | 1 | 57.00 | *FAK^6,7^* | N | RT-PCR/ IHC/Basescope |
|  | 43 | 62 | M | Yes | 2 | 24.20 | *FAK^6,7^* | A | RT-PCR/ IHC |
|  | 44 | 51 | F | Yes | 1 | 69.23 | *FAK^6,7^* | A | RT-PCR/ IHC/Basescope |
|  | 45 | 66 | F | No | 1 |  | *FAK* | Y | RT-PCR/ IHC/Basescope |
|  | 46 | 60 | M | Yes | 3 | 11.47 | *FAK* | P | RT-PCR/ IHC |
|  | 47 | 48 | F | No | 2 | 43.50 | *FAK^6,7^* | N | RT-PCR/ IHC/Basescope |
|  | 48 | 57 | M | Yes | 3 | 14.50 | *FAK* | Y | RT-PCR/ IHC |
|  | 49 | 64 | F | Yes | 2 |  | *FAK^6,7^* | A | RT-PCR/ IHC/Basescope |
|  | 50 | 62 | F | No | 2 |  | *FAK^6,7^* | A | RT-PCR/ IHC/Basescope |
|  | 51 | 68 | M | No | 1 | 9.77 | *FAK^6^/FAK^7^* | A | RT-PCR/ IHC/Basescope |
|  | 52 | 49 | M | Yes | 2 |  | *FAK* | N | RT-PCR/ IHC/Basescope |
|  | 53 | 49 | M | Yes | 2 | 36.47 | *FAK^6,7^* | N | RT-PCR/ IHC/Basescope |
|  | 54 | 51 | M | Yes | 1 | 75.33 | *FAK^6,7^* | A | RT-PCR/ IHC |
|  | 55 | 67 | M | Yes | 1 |  | *FAK^6,7^* | N | RT-PCR/ IHC/Basescope |
|  | 56 | 63 | M | Yes | 2 | 14.93 | *FAK* | Y | RT-PCR/ IHC |
|  | 57 | 62 | M | Yes | 2 |  | *FAK* | A | RT-PCR/ IHC/Basescope |
|  | 58 | 44 | F | No | 3 | 93.43 | *FAK* | N | RT-PCR/ IHC/Basescope |
|  | 59 | 48 | F | No | 1 | 73.00 | *FAK^6,7^* | N | RT-PCR/ IHC |
|  | 60 | 50 | M | Yes | 3 |  | *FAK* | Y | RT-PCR/ IHC/Basescope |
|  | 61 | 49 | F | No | 2 |  | *FAK* | Y | RT-PCR/ IHC/Basescope |
|  | 62 | 55 | M | Yes | 2 | 74.63 | *FAK* | Y | RT-PCR/ IHC/Basescope |
|  | 63 | 60 | M | Yes | 1 | 46.07 | *FAK^6,7^* | N | RT-PCR/ IHC/Basescope |
|  | 64 | 48 | M | Yes | 3 | 39.53 | *FAK^6^/FAK^7^* | N | RT-PCR/ IHC/Basescope |
|  | 65 | 55 | M | Yes | 1 | 67.23 | *FAK* | A | RT-PCR/ IHC |
|  | 66 | 57 | M | Yes | 3 |  | *FAK^6,7^* | A | RT-PCR/ IHC/Basescope |
|  | 67 | 54 | M | Yes | 1 | 4.40 | *FAK^6,7^* | A | RT-PCR/ IHC/Basescope |
|  | 68 | 62 | F | Yes | 3 | 52.60 | *FAK^6,7^* | N | RT-PCR/ IHC |
|  | 69 | 41 | F | No | 3 |  | *FAK^6,7^* | N | RT-PCR/ IHC/Basescope |
|  | 70 | 61 | F | No | 1 | 33.47 | *FAK^6,7^* | A | RT-PCR/ IHC/Basescope |
|  | 71 | 56 | M | Yes | 3 | 7.03 | *FAK^6,7^* | A | RT-PCR/ IHC |
|  | 72 | 68 | F | Yes | 1 | 52.57 | *FAK^6,7^* | N | RT-PCR/ IHC |
|  | 73 | 66 | M | Yes | 1 | 51.37 | *FAK^6,7^* | N | RT-PCR/ IHC/Basescope |
|  | 74 | 60 | F | No | 3 | 21.10 | *FAK^6,7^* | A | RT-PCR/ IHC |
|  | 75 | 49 | F | No | 1 | 70.90 | *FAK^6,7^* | N | RT-PCR/ IHC/Basescope |
|  | 76 | 54 | M | Yes | 2 | 71.87 | *FAK^6,7^* | A | RT-PCR/ IHC/Basescope |
|  | 77 | 64 | M | Yes | 3 |  | *FAK^6,7^* | N | RT-PCR/ IHC/Basescope |
|  | 78 | 49 | M | No | 1 | 58.27 | *FAK^6,7^* | A | RT-PCR/ IHC/Basescope |
|  | 79 | 62 | M | Yes | 3 | 61.53 | *FAK^6,7^* | A | RT-PCR/ IHC/Basescope |
|  | 80 | 63 | F | Yes | 3 | 82.27 | *FAK^6^/FAK^7^* | N | RT-PCR/ IHC |
|  | 81 | 49 | F | Yes | 1 |  | *FAK^6,7^* | A | RT-PCR/ IHC |
|  | 82 | 61 | F | Yes | 1 | 81.70 | *FAK^6,7^* | A | RT-PCR/ IHC |
|  | 83 | 59 | M | Yes | 1 | 32.83 | *FAK^6,7^* | N | RT-PCR/ IHC |
|  | 84 | 50 | M | No | 1 | 26.03 | *FAK^6^/FAK^7^* | N | RT-PCR/ IHC |
|  | 85 | 58 | M | No | 1 | 42.03 | *FAK^6,7^* | A | RT-PCR/ IHC/Basescope |
|  | 86 | 62 | F | Yes | 1 | 18.93 | *FAK^6,7^* | A | RT-PCR/ IHC/Basescope |
|  | 87 | 44 | M | Yes | 1 | 86.87 | *FAK* | Y | RT-PCR/ IHC/Basescope |
|  | 88 | 62 | M | Yes | 3 | 61.53 | *FAK* | Y | RT-PCR/ IHC/Basescope |
|  | 89 | 60 | M | No | 4 |  | *FAK* | N | RT-PCR/ IHC/Basescope |
|  | 90 | 54 | M | Yes | 3 | 37.60 | *FAK^6,7^* | A | RT-PCR/ IHC/Basescope |
|  | 91 | 54 | M | Yes | 1 | 26.00 | *FAK^6,7^* | A | RT-PCR/ IHC/Basescope |
|  | 92 | 54 | F | No | 3 |  | *FAK* | P | RT-PCR/ IHC |
|  | 93 | 58 | M | No | 2 | 12.20 | *FAK^6,7^* | A | RT-PCR/ IHC |
|  | 94 | 49 | F | No | 1 | 103.97 | *FAK^6,7^* | A | RT-PCR/ IHC/Basescope |
|  | 95 | 44 | M | Yes | 2 | 14.93 | *FAK^6^/FAK^7^* | A | RT-PCR/ IHC |
|  | 96 | 60 | M | Yes | 3 | 47.17 | *FAK* | Y | RT-PCR/ IHC |
|  | 97 | 61 | M | Yes | 3 | 20.67 | *FAK^6,7^* | A | RT-PCR/ IHC/Basescope |
|  | 98 | 61 | M | Yes | 3 | 12.17 | *FAK* | Y | RT-PCR/ IHC/Basescope |
|  | 99 | 46 | F | No | 2 | 76.33 | *FAK^6,7^* | A | RT-PCR/ IHC/Basescope |
|  | 100 | 59 | F | No | 3 | 15.83 | *FAK* | Y | RT-PCR/ IHC |
|  | 101 | 63 | F | Yes | 1 | 63.90 | *FAK^6,7^* | N | RT-PCR/ IHC/Basescope |
|  | 102 | 77 | F | No | 2 |  | *FAK^6,7^* | N | RT-PCR/ IHC |
|  | 103 | 34 | M | Yes | 2 |  | *FAK^6,7^* | A | RT-PCR/ IHC |
|  | 104 | 55 | F | No | 1 | 8.47 | *FAK^6,7^* | A | RT-PCR/ IHC |
|  | 105 | 58 | F | Yes | 2 | 83.00 | *FAK^6,7^* | A | RT-PCR/ IHC/Basescope |
|  | 106 | 44 | F | No | 3 | 32.73 | *FAK^6,7^* | A | RT-PCR/ IHC/Basescope |
|  | 107 | 58 | M | Yes | 3 | 26.43 | *FAK^6,7^* | N | RT-PCR/ IHC |
|  | 108 | 51 | M | Yes | 1 | 57.97 | *FAK^6,7^* | N | RT-PCR/ IHC/Basescope |
|  | 109 | 64 | M | Yes | 1 | 62.67 | *FAK* | Y | RT-PCR/ IHC |
|  | 110 | 42 | M | Yes | 3 | 32.27 | *FAK^6,7^* | Y | RT-PCR/ IHC |
|  | 111 | 48 | M | No | 1 | 73.57 | *FAK* | Y | RT-PCR/ IHC |
|  | 112 | 45 | F | Unknown | 1 | 59.60 | *FAK^6,7^* | N | RT-PCR/ IHC |
|  | 113 | 59 | F | No | 3 | 23.67 | *FAK^6,7^* | N | RT-PCR/ IHC/Basescope |
|  | 114 | 40 | M | Yes | 1 | 93.23 | *FAK^6,7^* | A | RT-PCR/ IHC/Basescope |
|  | 115 | 55 | M | Yes | 1 | 66.10 | *FAK* | Y | RT-PCR/ IHC |
|  | 116 | 34 | F | No | 3 | 24.90 | *FAK^6,7^* | N | RT-PCR/ IHC |
|  | 117 | 71 | M | Yes | 1 |  | *FAK^6^/FAK^7^* | P | RT-PCR/ IHC/Basescope |
|  | 118 | 44 | M | Yes | 1 |  | *FAK^6,7^* | N | RT-PCR/ IHC/Basescope |
|  | 119 | 62 | M | No | 1 | 18.70 | *FAK^6^/FAK^7^* | P | RT-PCR/ IHC |
|  | 120 | 56 | M | Yes | 4 |  | *FAK^6,7^* | A | RT-PCR/ IHC/Basescope |
|  | 121 | 65 | F | Yes | 1 | 49.57 | *FAK^6^/FAK^7^* | A | RT-PCR/ IHC |
|  | 122 | 49 | M | Yes | 1 | 102.27 | *FAK^6,7^* | N | RT-PCR/ IHC |
|  | 123 | 55 | M | No | 2 | 11.80 | *FAK^6,7^* | A | RT-PCR/ IHC |
|  | 124 | 46 | M | Yes | 1 | 26.20 | *FAK^6,7^* | N | RT-PCR/ IHC |
|  | 125 | 62 | M | Yes | 1 | 14.83 | *FAK^6^/FAK^7^* | A | RT-PCR/ IHC |
|  | 126 | 59 | F | No | 2 | 53.23 | *FAK* | Y | RT-PCR/ IHC/Basescope |
|  | 127 | 52 | F | No | 2 | 35.20 | *FAK* | Y | RT-PCR/ IHC |
|  | 128 | 53 | F | No | 3 | 42.70 | *FAK* | Y | RT-PCR/ IHC |
|  | 129 | 48 | M | Yes | 1 | 49.57 | *FAK* | N | RT-PCR/ IHC |
|  | 130 | 51 | M | Yes | 2 | 56.37 | *FAK^6,7^* | A | RT-PCR/ IHC/Basescope |
|  | 131 | 68 | M | No | 3 | 9.33 | *FAK^6,7^* | A | RT-PCR/ IHC |
|  | 132 | 65 | M | Yes | 3 |  | *FAK^6^/FAK^7^* | N | RT-PCR/ IHC/Basescope |
|  | 133 | 56 | M | Yes | 1 | 64.30 | *FAK* | P | RT-PCR/ IHC/Basescope |
|  | 134 | 65 | M | Yes | 2 |  | *FAK^6,7^* | A | RT-PCR/ IHC |
|  | 135 | 72 | M | Yes | 3 | 9.77 | *FAK^6,7^* | A | RT-PCR/ IHC/Basescope |
|  | 136 | 52 | F | No | 3 |  | *FAK^6,7^* | A | RT-PCR/ IHC/Basescope |
| Frozen tissue | 137 | 53 | M | YES | NA. |  | *FAK^6^/FAK^7^* | N | RNA-seq |
|  | 138 | 60 | M | YES | NA. | 35.86667 | *FAK^6^/FAK^7^* | A | RNA-seq |
|  | 139 | 47 | M | YES | NA. | 13.03 | *FAK^6^/FAK^7^* | A | RNA-seq |
|  | 140 | 58 | M | YES | NA. | 23.4 | *FAK* | Y | RNA-seq |
|  | 141 | 53 | M | YES | NA. |  | *FAK^6^/FAK^7^* | N | RNA-seq |
|  | 142 | 66 | M | YES | NA. | 15.77 | *FAK^6^/FAK^7^* | A | RNA-seq |
|  | 143 | 57 | M | YES | NA. | 31.77 | *FAK^6^/FAK^7^* | A | RNA-seq |
|  | 144 | 61 | M | YES | NA. | 33 | *FAK^6^/FAK^7^* | A | RNA-seq |
|  | 145 | 84 | M | YES | NA. | 21.46667 | *FAK^6^/FAK^7^* | Y | RNA-seq |
|  | 146 | 66 | F | NO | NA. | 25.2 | *FAK^6^/FAK^7^* | A | RNA-seq |
|  | 147 | 78 | M | YES | NA. | 2.43 | *FAK^6^/FAK^7^* | N | RNA-seq |
|  | 148 | 57 | M | YES | NA. | 9.8 | *FAK^6^/FAK^7^* | A | RNA-seq |
|  | 149 | 65 | M | YES | NA. | 3.77 | *FAK^6^/FAK^7^* | P | RNA-seq |
|  | 150 | 68 | M | YES | NA. | 64.73333 | *FAK^6^/FAK^7^* | P | RNA-seq |
|  | 151 | 68 | M | YES | NA. |  | *FAK^6^/FAK^7^* | N | RNA-seq |
|  | 152 | 68 | M | YES | NA. | 42.46667 | *FAK^6^/FAK^7^* | A | RNA-seq |
|  | 153 | 45 | M | NO | NA. | 35.13333 | *FAK^6^/FAK^7^* | N | RNA-seq |
|  | 154 | 61 | M | YES | NA. | 60.83333 | *FAK* | Y | RNA-seq |

ANPY subtype: A, ASCL1; N, NeuroD1; P, Pou2F3; Y, YAP. Sex: F, female; M, male.

**Table S11. *FAK* in each of the 154 patients.**

Table S12.

| Type | wt FAK, n (%) | FAK^6/^FAK^7^, n (%) | FAK^6,7^, n (%) | FAK^S^, n (%) | Total | *P** |
| --- | --- | --- | --- | --- | --- | --- |
| *ASCL1* | 3 (8.6) | 13 (43.3) | 52 (58.4) | 65 (95.6) | 68 | 8.2626E-18 |
| *NEUROD1* | 5 (14.3) | 10 (33.3) | 35 (39.3) | 45 (90) | 50 |  |
| *POU2F3* | 6 (17.1) | 5 (16.7) | 1 (1.1) | 6 (50) | 12 |  |
| *YAP* | 21 (60.0) | 2 (6.7) | 1 (1.1) | 3 (12.5) | 24 |  |
| Total | 35 | 30 | 89 | 119 | 154 |  |

wt, wild-type. *, [Chi-square test](https://blog.csdn.net/u011955252/article/details/50704459).

**Table S12. *FAK* splicing alternatives in the 4 subtypes of SCLC.**

Table S16.

| REAGENT or RESOURCE | SOURCE | IDENTIFIER |
| --- | --- | --- |
| Antibodies | | |
| anti-mouse/human FAK (polyclonal) | Cell Signaling Technology | cat#3285; RRID: AB_2269034 |
| anti- human Phospho-FAK (Tyr397) (Clone D20B1) | Cell Signaling Technology | cat#8556; RRID: AB_10891442 |
| anti- mouse/human β-Actin (clone AC-15) | Sigma | cat#A5441; RRID: AB_476744 |
| anti- mouse/human Phospho-FAK (Tyr397) (clone 31H5L17 ) | Thermo Fisher Scientific | cat#700255; RRID: AB_2532307 |
| anti- mouse/human Phospho-Mtor (Ser2448) (clone D9C2) | Cell Signaling Technology | cat#5536; RRID: AB_10691552 |
| anti- mouse/human mTOR (clone 7C10) | Cell Signaling Technology | cat#2983; RRID: AB_2105622 |
| anti- mouse/human Phospho-p70 S6 Kinase (Thr389) (clone 108D2) | Cell Signaling Technology | cat#9234; RRID: AB_2269803 |
| anti- human Phospho-AKT (Ser473) (polyclonal) | Santa Cruz Biotechnology | cat#sc-101629; RRID: AB_2224735 |
| anti- human AKT (polyclonal) | Santa Cruz Biotechnology | cat#sc-8312; RRID: AB_671714 |
| anti- mouse/human Ki67 (polyclonal) | Abcam | cat#ab15580; RRID: AB_443209 |
| anti-human IFN-γ (clone AKYP0093) | Akoya Biosciences | cat#4250062 |
| anti-human CD21 (clone AKYP0061) | Akoya Biosciences | cat#4450027 |
| anti-human MPO (clone AKYP0113) | Akoya Biosciences | cat#4250083; RRID: AB_2927678 |
| anti-human TOX (clone AKYP0098) | Akoya Biosciences | cat#4250067 |
| anti-human Granzyme B (clone AKYP0086) | Akoya Biosciences | cat#4250055 |
| anti-human CD8 (clone AKYP0028) | Akoya Biosciences | cat#4250012; RRID: AB_2915960 |
| anti-human CD45 (clone AKYP0074) | Akoya Biosciences | cat#4250099 |
| anti-human CD45RO (clone AKYP0059) | Akoya Biosciences | cat#4550127 |
| anti-human LAG3 (clone AKYP0089) | Akoya Biosciences | cat#4550058; RRID: AB_3096409 |
| anti-human CD56 (clone AKYP0118) | Akoya Biosciences | cat#4550126 |
| anti-human CD3E (clone AKYP0062) | Akoya Biosciences | cat#4550119; RRID: AB_2936080 |
| anti-human FoxP3 (clone AKYP0102) | Akoya Biosciences | cat#4550071; RRID: AB_2927679 |
| anti-human PD-1 (clone AKYP0070) | Akoya Biosciences | cat#4550038; RRID: AB_3096407 |
| anti-human CD68 (clone AKYP0050 ) | Akoya Biosciences | cat#4550113; RRID: AB_2935894 |
| anti-human CD4 (clone AKYP0048) | Akoya Biosciences | cat#4550112; RRID: AB_3094499 |
| anti-human EpCAM (clone AKYP0119) | Akoya Biosciences | cat#4450088 |
| anti-human HLA-A (clone AKYP0078) | Akoya Biosciences | cat#4450046 |
| anti-human CD79a (clone AKYP0109) | Akoya Biosciences | cat#4450078; RRID: AB_3082977 |
| anti-human Pan-Cytokeratin (clone AKYP0053) | Akoya Biosciences | cat#4450093 |
| anti-human HLA-DR (clone AKYP0063) | Akoya Biosciences | cat#4450095; RRID: AB_3094500 |
| anti-mouse/human Ki-67 (clone AKYP0052) | Akoya Biosciences | cat#4450096; RRID: AB_3094497 |
| anti-human Vimentin (clone AKYP0082) | Akoya Biosciences | cat#4450050; RRID: AB_2935889 |
| anti-human CD20 (clone AKYP0049) | Akoya Biosciences | cat#4450094; RRID: AB_3094498 |
| anti-human CD14 (clone EPR3653) | Abcam | cat#ab226121 |
| anti-human PD-L1 (clone 73-10) | Abcam | cat#ab226766; RRID: AB_3073663 |
| Biological samples |  |  |
| Primary SCLC tumor samples | Cancer Hospital Chinese Academy of Medical Sciences; the Third Affiliated Hospital of Kunming Medical University; Sun Yat-Sen University Cancer Center; Tianjin Medical University Cancer Institute and Hospital; Peking University Cancer Hospital and Institute; Ruijin Hospital Affiliated to Shanghai Jiao Tong University School of Medicine; Guangzhou Provincial People Hospital; The Second Affiliated Hospital Zhejiang University School of Medicine; Affiliated Hospital, School of Clinical Medicine, Tangshan | N/A |
| Primary normal lung samples | Cancer Hospital Chinese Academy of Medical Sciences; the Third Affiliated Hospital of Kunming Medical University; Sun Yat-Sen University Cancer Center; Tianjin Medical University Cancer Institute and Hospital; Peking University Cancer Hospital and Institute; Ruijin Hospital Affiliated to Shanghai Jiao Tong University School of Medicine; Guangzhou Provincial People Hospital; The Second Affiliated Hospital Zhejiang University; Affiliated Hospital, School of Clinical Medicine School of Medicine, Tangshan | N/A |
| SCLC Blood samples | Affiliated Hospital, School of Clinical Medicine, Tangshan; The Second Affiliated Hospital of Zhejiang University School of Medicine; | N/A |
| SCLC formalin-fixed paraffin-embedded (FFPE) samples | Department of Internal Medicine, Harbin Medical University Cancer Center | N/A |
| PDX1 of SCLC | BEIJING IDMO Co., Ltd | cat#17102417E05T |
| PDX2 of SCLC | BEIJING IDMO Co., Ltd | cat#17120501E05T |
| Chemicals, peptides, and recombinant proteins | | |
| RPMI 1640 | Gibco | cat#31800-014 |
| DMEM | Gibco | cat#12800-017 |
| Fetal Bovine Serum | Gibco | cat#10099158 |
| DMSO | MP | cat#196055 |
| BSA | Roche | cat#AP0027 |
| 0.25% Trypsin-EDTA | Gibco | cat#25200-72 |
| Tween-20 | Amresco Inc. | cat#0777 |
| DAPI | Incitrogen | cat#2305156 |
| TEMED | Solarbio | cat#T8090 |
| NCM Universal Antibody Diluent | NCM Biotech | cat#WB500D |
| Ncm ECL Ultra | NCM Biotech | cat#P10300 |
| Triton X-100 | Solarbio | cat#T8200 |
| Acryl/Bis 30% Solution (29:1) | Sangon Biotech | cat#B546017 |
| PVDF membrane | Millipore | cat#IPVH00010 |
| Protein Ladder | ThermoFisher | cat#26616 |
| SDS | JiSiEnBei | cat#JS0054-500G |
| Phosphatase inhibitors | Bimake | cat#B15001 |
| Penicillin-Streptomycin 100× solution | Gibco | cat#15140-122 |
| Trypsin | Gibco | cat#25200-72 |
| collagenase I | Gibco | cat#17100–017 |
| collagenase IV | Gibco | cat#17104–019 |
| DMEM/F12 | Gibco | cat#C11330500BT |
| Matrigel | Corning | cat#356237 |
| BX006 | Akoya Biosciences | cat#5550018 |
| BX027 | Akoya Biosciences | cat#5550011 |
| EDTA antigen retrieval solution (50×) | WEIAOBIO | cat#WH1034 |
| 16% Formaldehyde Solution (W/V),Methanol-free | ThermoFisher | cat#28908 |
| Dimethyl sulfoxide | Sigma-Aldrich | cat#472301-4L |
| Trypan Blue powder | Sigma-Aldrich | cat#T6146 |
| Ammonium persulfate | BioMOL | cat#H8N208S2 |
| TotalSeq™-A Human Universal Cocktail | Biolegend | cat#399907 |
| Protease Inhibitor Cocktail | Roche | cat#04693159001 |
| Yeast extract | OXOID. | cat#LP0021 |
| Tryptone | OXOID | cat#LP0042 |
| Tween-20 | Amresco Inc. | cat#0777 |
| DAPI | Incitrogen | cat#2305156 |
| protein A/G agarose | Santa Cruz Biotechnology | cat#sc-2003 |
| TRIzol Reagent | Invitrogen | cat#15596018 |
| PF-562271 | Selleck | cat#S2890 |
| PF-573228 | Selleck | cat#S2013 |
| Torin1 | Selleck | cat#S2827 |
| MK-2206 2HCL | Selleck | cat#S1078 |
| Phosphate-buffered saline | HyClone | cat#SH30256.12 |
| Critical commercial assays | | |
| Cell Counting Kit-8 | Vazyme | cat#A311-01 |
| DNA Gel Extraction Kit | Axygen | cat#AP-GX-50 |
| eECL Western Blot Kit | CWBIO | cat#CW0049M |
| Antibody Conjugation Kit | Akoya Biosciences | cat#7000009 |
| 10X Buffer Kit for PhenoCycler-Fusion | Akoya Biosciences | cat#7000019 |
| Assay Reagent for PhenoCycler | Akoya Biosciences | cat#7000002 |
| Nuclear Stain for PhenoCycler | Akoya Biosciences | cat#7000003 |
| Sample Kit for PhenoCycler-Fusion | Akoya Biosciences | cat#7000017 |
| Agilent SureSelect Human All Exon V6 Kit | Agilent | cat#5190-8865 |
| sCelLiVE® Tissue Dissociation Kit | Singleron | cat#1190062 |
| GEXSCOPE® Single Cell RNA Library Kits | Singleron | cat#5180031 |
| NEBNext Ultra™ RNA Library Prep Kit for Illumina® | New England Biolabs | cat#E7530 |
| Femto Bacterial DNA Quantification Kit | Zymo Research | cat#E2006 |
| TaqMan™ Fast Advanced Master Mix | Thermo Fisher Scientific | cat#4444556 |
| AllPrep DNA/RNA Mini Kit | Qiagen | cat#80204 |
| RNeasy FFPE Kit | Qiagen | cat#73504 |
| Phusion High-Fidelity PCR Master Mix | New England Biolabs | cat#M0531S |
| GeneJET Gel Extraction Kit | Thermo Fisher Scientific | cat#K0691 |
| Ion Plus Fragment Library Kit | Thermo Fisher Scientific | cat#4471252 |
| MultiS One-step Cloning Kit | Vazyme | cat#C113 |
| HiScript III RT SuperMix for qPCR (+gDNA wiper) | Vazyme | cat#R323 |
| Basescope Duplex Regent Kit | Advanced Cell Diagnostics | cat#323800 |
| Magnetic Luminex® Assay | R&D Systems | cat#LXSAH |
| Universal Tyrosine Kinase Assay Kit | Clontech | cat#MK410 |
| 2 × Rapid Taq Master Mix | Vazyme | cat#P222 |
| 5 min TA/Blunt-Zero Cloning Kit | Vazyme | cat#601 |
| HiFiScript cDNA Synthesis Kit | Vazyme | cat#R312 |
| SYBR® Premix Ex Taq™ | Vazyme | cat#Q712 |
| Plasmid Midi Preparation Kit | CWBio | cat#CW21055 |
| Lipo3000 Kit | Invitrogen | cat#L3000-015 |
| Deposited data | | |
| Human SCLC scRNA-seq data | this paper | National Genomics Data Center of China, PRJCA023692,HRA007131, HRA007382 |
| Human SCLC WES data | this paper | National Genomics Data Center of China, PRJCA024141,  HRA007141 |
| Human SCLC bulk RNA-seq data | this paper | National Genomics Data Center of China, PRJCA024130,  HRA007125 |
| Human SCLC 16S rRNA gene-seq data | this paper | National Genomics Data Center of China, PRJCA025292,CRA016047 |
| Human SCLC scRNA-seq data | Wu et al., 2021 | GEO accession: GSE148071 |
| Human SCLC scRNA-seq data | Hu et al., 2023 | GEO accession: GSE207422 |
| Human SCLC bulk RNA-seq data | Chen et al., 2021 | ENA Database: PRJEB42904 |
| Human SCLC bulk RNA-seq data | George et al., 2015 | EGA Database: EGAS00001000925 |
| Human SCLC bulk RNA-seq data | Jiang et al., 2016 | GEO accession: GSE60052 |
| Human SCLC WES data | Chen et al., 2021 | ENA Database: PRJEB42904 |
| Human SCLC WES data (Caucasian) | George et al., 2015 | EGA Database: EGAS00001000925 |
| Human SCLC WES data (Caucasian) | Rudin et al., 2012 | EGA Database:  EGAS00001000334 |
| Experimental models: Cell lines | | |
| DMS114 | ATCC | cat#CRL-2066; RRID: CVCL_1174 |
| DMS153 | ATCC | cat#CRL-2064; RRID: CVCL_1175 |
| H69 | ATCC | cat#HTB-119; RRID: CVCL_1579 |
| H82 | ATCC | cat#HTB-175; RRID: CVCL_1591 |
| H446 | ATCC | cat#HTB-171; RRID: CVCL_1562 |
| H524 | ATCC | cat#CRL-5831; RRID: CVCL_1568 |
| H2227 | ATCC | cat#CRL-5934; RRID: CVCL_1542 |
| H1339 | ATCC | cat#CRL-5979; RRID: CVCL_A472 |
| H1688 | ATCC | cat#CCL-257; RRID: CVCL_1487 |
| Calu-6 | ATCC | cat#HTB-56; RRID: CVCL_0236 |
| 16HBE | Merck | cat#SCC150,  RRID: CVCL_0112 |
| HEK293 | ATCC | cat#CRL-1573; RRID: CVCL_0045 |
| Experimental models: Organisms/strains | | |
| Mouse: nonobese diabetic (NOD)/ShiLtJGpt-Prkdc^em26Cd52^Il2rg^em26Cd22^/Gpt (null; NCG) | GemPharmatech | cat#T001475 |
| Oligonucleotides | | |
| Primer: *FAK-Plus (E14-E16):*  Forward (5’ to 3’): AAGCAAGGCATGCGGACACA  Reverse (5’ to 3’): CTCTTTGAATCTCATAATCCCTGG | this paper | N/A |
| Primer: *FAK (E10-E18):*  Forward (5’ to 3’): TAACGGACAAGGGCTGCAAT  Reverse (5’ to 3’): AAGTGTGCACAGCTCCATGA | this paper | N/A |
| Primer: *FAK (*transcript*):*  Forward (5’ to 3’): GATGACAAACTTAAGGAATTCATGGCAGCTGCTTAC  Reverse (5’ to 3’): CGAGGAATCGGGCCCGGATCCTCAGTGTGGTCTCGT | this paper | N/A |
| Primer: *vREST:*  Forward (5’ to 3’): AGAACGCCCATATAAATGTGAACTTT  Reverse (5’ to 3’): GGCGGGTTACTTCATGTTGATT | this paper | N/A |
| Primer: *CSNK1D-CCDC57:*  Forward (5’ to 3’): AGCCGGGAGCCGGACC  Reverse (5’ to 3’): TGGCTTTGGGGACCTGTCTTCTTT | this paper | N/A |
| Primer: *ARID1B-ZDHHC14:*  Forward (5’ to 3’): AATGCAGTACCCTCAGCAGCA  Reverse (5’ to 3’): CGAGGTTGGGCATGGAGGCG | this paper | N/A |
| Primer: *β-Actin:*  Forward (5’ to 3’): TCAACACCCCAGCCATGTAC  Reverse (5’ to 3’): AGGGCATACCCCTCGTAGAT | this paper | N/A |
| Primer: 515FB-806RB*:*  Forward (5’ to 3’): GTGYCAGCMGCCGCGGTAA  Reverse (5’ to 3’): GGACTACNVGGGTWTCTAAT | this paper | N/A |
| Primer: *CCL1:*  Forward (5’ to 3’): GCCCAAGCCAGACCAGAAGACA  Reverse (5’ to 3’): AAGCAGGGCAGAAGGAATGGTG | this paper | N/A |
| Primer: *CCL5:*  Forward (5’ to 3’): ACCATGAAGGTCTCCGCG  Reverse (5’ to 3’): TTCAGGTTCAAGGACTCTCCA | this paper | N/A |
| Primer: *CCL7:*  Forward (5’ to 3’): AGACCAAACCAGAAACCTCC  Reverse (5’ to 3’): AGTATTAATCCCAACTGGCTGAG | this paper | N/A |
| Primer: *CXCL10:*  Forward (5’ to 3’): TCTGAATCCAGAATCGAAGG  Reverse (5’ to 3’): CTCTGTGTGGTCCATCCTTG | this paper | N/A |
| Primer: *CXCL13:*  Forward (5’ to 3’): AGTCTGAACTCTACCTCCAG  Reverse (5’ to 3’): TTGTGTAATAGACCTCCAGA | this paper | N/A |
| Primer: *BCL2:*  Forward (5’ to 3’): AGTACCTGAACCGGCACCTG  Reverse (5’ to 3’): CATGCTGGGGCCGTATCGTT | this paper | N/A |
| Primer: *IL6:*  Forward (5’ to 3’): CCACTCACCTCTTCAGAACGAATT  Reverse (5’ to 3’): AGTGCCTCTTTGCTGCTTTCA | this paper | N/A |
| Primer: *IL33:*  Forward (5’ to 3’): AGCAAAGTGGAAGAACACAGC  Reverse (5’ to 3’): CTTCTTTGGCCTTCTGTTGG | this paper | N/A |
| Primer: *MDM2:*  Forward (5’ to 3’): AGGAGATTTGTTTGGCGTGC  Reverse (5’ to 3’): TGAGTCCGATGATTCCTGCTG | this paper | N/A |
| Primer: *MYC:*  Forward (5’ to 3’): GGCTCCTGGCAAAAGGTCA  Reverse (5’ to 3’):CTGCGTAGTTGTGCTGATGT | this paper | N/A |
| Primer: *GAPDH:*  Forward (5’ to 3’): CGGAGTCAACGGATTTGGTCGTAT  Reverse (5’ to 3’): AGCCTTCTCCATGGTGGTGAAGAC | this paper | N/A |
| siRNA: si*FAK*-1  sense: GCCCAGGUUUACUGAACUUAAdTdT  antisense: UUAAGUUCAGUAAACCUGGGCdTdT | this paper | N/A |
| siRNA:si*FAK*-2  sense: CAACAGGUGAAGAGCGAUUAUdTdT  antisense: AUAAUCGCUCUUCACCUGUUGdTdT | this paper | N/A |
| Recombinant DNA | | |
| pcDNA3.1-Flag-NC | this paper | N/A |
| pcDNA3.1-Flag-FAK^wt^ | this paper | N/A |
| pcDNA3.1-Flag-FAK^6^ | this paper | N/A |
| pcDNA3.1-Flag-FAK^7^ | this paper | N/A |
| pcDNA3.1-Flag-FAK^6,7^ | this paper | N/A |
| Software and algorithms | | |
| R v4.13 | N/A | <https://www.r-project.org/> |
| BWA v0.1.22 | Li et al., 2009 | <http://bio-bwa.sourceforge.net/>  RRID: SCR_010910 |
| Picard v1.4.5 | N/A | <http://broadinstitute.github.io/picard/>  RRID: SCR_006525 |
| MuTect v1.1.4 | N/A | <http://www.broadinstitute.org/cancer/cga/mutect>  RRID: SCR_000559 |
| Strelka v1.0.13 | Saunders et al., 2012 | <https://github.com/Illumina/strelka>  RRID: SCR_005109 |
| MutSigCV v1.41 | Lawrence et al., 2013 | <https://www.genepattern.org/modules/docs/MutSigCV> |
| MutationalPatterns v1.8.0 | Blokzijl et al., 2018 | <https://bioconductor.org/packages/release/bioc/html/MutationalPatterns.html>  RRID: SCR_024247 |
| Palimpsest v2.0.0 | Shinde et al., 2018 | <https://github.com/FunGeST/Palimpsest> |
| CeleScope v 1.9.0 | Singleron | <https://github.com/singleron-RD/CeleScope>  RRID: SCR_023553 |
| Seurat v 4.0 | Butler et al., 2018^,^ ;Butler et al., 2019 | <http://seurat.r-forge.r-project.org/>  RRID: SCR_007322 |
| TopGO v 3.13 | N/A | <http://bioconductor.org/packages/release/bioc/html/topGO.html>  RRID: SCR_014798 |
| GSEA v4.0.3 | Subramanian et al., 2005 | <http://www.broadinstitute.org/gsea/>  RRID: SCR_003199 |
| Yeskit | Zhang et al., 2022 | <https://github.com/ncrna/Yeskit> |
| CytoTRACE2 | Kang et al., 2024; Gulati et al., 2020 | [GitHub - digitalcytometry/cytotrace2: CytoTRACE 2 is an interpretable AI method for predicting cellular potency and absolute developmental potential from scRNA-seq data.](https://github.com/digitalcytometry/cytotrace2) |
| CellChat | NA | <https://github.com/sqjin/CellChat>  RRID:SCR_021946 |
| Hisat2 v2.0.5 | Kim et al., 2019 | <http://ccb.jhu.edu/software/hisat2/index.shtml>  RRID: SCR_015530 |
| featureCounts v1.5.0 | Liao et al., 2014 | <https://subread.sourceforge.net/>  RRID: SCR_012919 |
| DESeq2 v1.16.1 | Love et al., 2014 | <https://bioconductor.org/packages/release/bioc/html/DESeq2.html>  RRID: SCR_015687 |
| clusterProfiler | Yu et al., 2012 | <http://bioconductor.org/packages/release/bioc/html/clusterProfiler.html>  RRID: SCR_016884 |
| ESTIMATE | Yoshihara et al, 2013 | <https://bioinformatics.mdanderson.org/estimate/rpackage.html> |
| GSVA | Hänzelmann et al.,2013 | <https://www.bioconductor.org/packages/release/bioc/html/GSVA.html>  RRID: SCR_021058 |
| CIBERSORT | Chen et al., 2018 | <https://cibersort.stanford.edu/>  RRID: SCR_016955 |
| xCell | Aran et al., 2017 | <http://xCell.ucsf.edu/> |
| STAR-Fusion v1.2.0 | Haas et al., 2019 | <https://github.com/STAR-Fusion/STAR-Fusion/wiki> |
| rMATS v4.0.1 | Shen et al., 2014 | <https://rmats.sourceforge.io>  RRID: SCR_023485 |
| STAR | Dobin et al., 2013 | <http://code.google.com/p/rna-star/>  RRID: SCR_004463 |
| AlphaFold Protein Structure Database | Varadi et al., 2022 | <https://alphafold.ebi.ac.uk/>  RRID: SCR_023662 |
| ENDscript | Robert et al., 2014 | <http://endscript.ibcp.fr> |
| PyMOL | DeLano et al., 2008 | <http://www.pymol.org/>  RRID:SCR_000305 |
| Cutadapt v1.9.1 | Kechin et al., 2017 | <http://code.google.com/p/cutadapt/>  RRID: SCR_011841 |
| UCHIME algorithm | Edgar et al., 2011 | <http://drive5.com/usearch/manual/uchime_algo.html>  RRID: SCR_008057 |
| Silva | Quast et al., 2013 | <http://www.arb-silva.de>  RRID: SCR_006423 |
| RDP Classifier v2.13 | Wang et al., 2007 | <https://sourceforge.net/projects/rdp-classifier/>  RRID: SCR_022773 |
| Phyloseq v1.30.0 | McMurdie et al., 2013 | <http://www.bioconductor.org/packages/2.12/bioc/html/phyloseq.html>  RRID: SCR_013080 |
| Vegan v2.5-7 | Oksanen et al., 2012 | <http://cran.r-project.org/web/packages/vegan/index.html>  RRID: SCR_011950 |
| QuPath | Bankhead et al., 2017 | <https://qupath.github.io/>  RRID: SCR_018257 |
| GraphPad Prism 5 | GraphPad | <http://www.graphpad.com/>  RRID: SCR_002798 |
| Other | | |
|  |  |  |

**Table S16. Key resources used in this study.**

**Uncropped images of Western Blots**

**References**

1	Hu, X. *et al.* TumorFusions: an integrative resource for cancer-associated transcript fusions. *Nucleic acids research* **46**, D1144-d1149, doi:10.1093/nar/gkx1018 (2018).

2	Liu, Q. *et al.* Proteogenomic characterization of small cell lung cancer identifies biological insights and subtype-specific therapeutic strategies. *Cell* **187**, 184-203.e128, doi:10.1016/j.cell.2023.12.004 (2024).

3	Ghandi, M. *et al.* Next-generation characterization of the Cancer Cell Line Encyclopedia. *Nature* **569**, 503-508, doi:10.1038/s41586-019-1186-3 (2019).

4	George, J. *et al.* Comprehensive genomic profiles of small cell lung cancer. *Nature* **524**, 47-53 (2015).

5	Jiang, L. *et al.* Genomic Landscape Survey Identifies SRSF1 as a Key Oncodriver in Small Cell Lung Cancer. *PLoS genetics* **12**, e1005895, doi:10.1371/journal.pgen.1005895 (2016).
